# Prevalence and Correlates of Dietary and Nutrition Information Seeking Through Various Web-Based and Offline Media Sources Among Japanese Adults: Web-Based Cross-Sectional Study

**DOI:** 10.2196/54805

**Published:** 2024-02-14

**Authors:** Kentaro Murakami, Nana Shinozaki, Tsuyoshi Okuhara, Tracy A McCaffrey, M Barbara E Livingstone

**Affiliations:** 1 Department of Social and Preventive Epidemiology School of Public Health The University of Tokyo Tokyo Japan; 2 Department of Nutritional Epidemiology and Behavioural Nutrition Graduate School of Medicine The University of Tokyo Tokyo Japan; 3 Department of Health Communication School of Public Health The University of Tokyo Tokyo Japan; 4 Department of Nutrition, Dietetics and Food Monash University Clayton Australia; 5 Nutrition Innovation Centre for Food and Health School of Biomedical Sciences Ulster University Coleraine United Kingdom

**Keywords:** nutrition, diet, information seeking, health literacy, food literacy, diet quality, Japan

## Abstract

**Background:**

The advent of the internet has changed the landscape of available nutrition information. However, little is known about people’s information-seeking behavior toward healthy eating and its potential consequences.

**Objective:**

We aimed to examine the prevalence and correlates of nutrition information seeking from various web-based and offline media sources.

**Methods:**

This cross-sectional study included 5998 Japanese adults aged 20 to 79 years participating in a web-based questionnaire survey (February and March 2023). The dependent variable was the regular use of web-based and offline media as a reliable source of nutrition information. The main independent variables included health literacy, food literacy, and diet quality, which were assessed using validated tools, as well as sociodemographic factors (sex, age, education level, and nutrition- and health-related occupations).

**Results:**

The top source of nutrition information was television (1973/5998, 32.89%), followed by web searches (1333/5998, 22.22%), websites of government and medical manufacturers (997/5998, 16.62%), newspapers (901/5998, 15.02%), books and magazines (697/5998, 11.62%), and video sites (eg, YouTube; 634/5998, 10.57%). Multivariable logistic regression showed that higher health literacy was associated with higher odds of using all the individual sources examined; odds ratios (ORs) for 1-point score increase ranged from 1.27 (95% CI 1.09-1.49) to 1.81 (95% CI 1.57-2.09). By contrast, food literacy was inversely associated with the use of television (OR 0.65, 95% CI 0.55-0.77), whereas it was positively associated with the use of websites of government and medical manufacturers (OR 1.98, 95% CI 1.62-2.44), books and magazines (OR 2.09, 95% CI 1.64-2.66), and video sites (OR 1.53, 95% CI 1.19-1.96). Furthermore, diet quality was positively associated with the use of newspapers (OR 1.02, 95% CI 1.01-1.03) and books and magazines (OR 1.03, 95% CI 1.02-1.04). Being female was associated with using television and books and magazines, whereas being male was associated with using websites of government and medical manufacturers, newspapers, and video sites. Age was positively associated with using newspapers and inversely associated with using websites of government and medical manufacturers and video sites. People with higher education were more likely to refer to websites of government and medical manufacturers and newspapers but less likely to use television and video sites. Dietitians were more likely to use websites of government and medical manufacturers and books and magazines than the general public but less likely to use television and video sites.

**Conclusions:**

We identified various web-based and offline media sources regularly used by Japanese adults when seeking nutrition information, and their correlates varied widely. A lack of positive associations between the use of the top 2 major sources (television and web searches) and food literacy or diet quality is highlighted. These findings provide useful insights into the potential for developing and disseminating evidence-based health promotion materials.

## Introduction

### Background

On a global scale, poor diet quality is a major risk factor for premature mortality and morbidity, accounting for 22% of total deaths and 15% of disability-adjusted life years annually; these estimates are even higher in East Asian countries, including Japan (30% and 21%, respectively) [1]. As diet is a key modifiable risk factor, it is not surprising that the focus on prevention of chronic diseases has become a priority [2], mainly by reducing the intake of foods high in sodium and added sugars (eg, ultraprocessed foods) [3] and increasing the intake of plant-based foods, including whole grains, nuts, legumes, fruits, and vegetables [4,5]. This robust evidence base connects food to human health and should satisfy the growing public demand for dietary and nutrition information.

However, the advent of the internet has permanently changed the landscape of information and its use [6,7]. In particular, the introduction of Web 2.0, in which people can create, edit, and share content, has irrevocably changed communication from that via initial static web pages (Web 1.0) and traditional media (eg, newspapers, television, and radio) [8,9]. For a large proportion of people, the internet has become an essential tool for improving health-related knowledge and behavior, including nutrition [10,11]. For example, a national survey estimated that 78% of Japan’s general population uses the internet, with 73% of them seeking health information [12]. However, owing to the lack of regulations and ease of creating and sharing web-based information, consumers have access to an abundance of web-based information of variable quality and accuracy [13,14]. Consequently, nutrition and health professionals increasingly struggle with disseminating evidence-based nutrition information in a web-based media landscape filled with unreliable and contradictory nutrition information (ie, misinformation) [14-17].

It is reasonable to assume that the level of health literacy (ie, the cognitive and social skills that determine an individual’s motivation and ability to access, understand, and use information in ways that promote and maintain health [18]) is probably related to the search for nutrition information and thus the acquisition of nutrition knowledge [19]. However, an appropriate level of health literacy does not automatically lead to proper nutrition knowledge, particularly regarding aspects related to correct dietary decision-making [20]. Consequently, increasing attention is now being directed toward food literacy, defined as “a collection of inter-related knowledge, skills and behaviors required to plan, manage, select, prepare and eat food to meet needs and determine intake” [21].

Information seeking rarely involves only 1 channel [22-24]. For example, people still value and use conventional (offline) sources of dietary and nutrition information [11,25-29]. This may be particularly the case in Japan, where, as consistently shown in previous studies, traditional media, such as television and newspapers, are still major sources of health-related information [30-34]. However, only a few Western studies have explored how profiles of dietary and nutrition information seekers vary by media source [11,28,29] or how the effects of dietary and nutrition information seeking vary by media source [25,26,35-38]. More importantly, a limited number of studies have assessed aspects of dietary habits as lifestyle behaviors (eg, fruit and vegetable consumption) using dietary assessment methods that were not fit for purpose [25,26,35-40]. What people eat is complex and notoriously difficult to measure [41] and particularly vulnerable to specific sources of bias, such as social desirability and social approval [42,43]. Furthermore, as foods cannot be eaten in isolation, diet quality, a composite measure of the intake of foods and nutrients considered important to health [44], can provide a more comprehensive and holistic measure of dietary behavior [45,46] and should, therefore, be better suited to the investigation of the potential consequences of dietary and nutrition information seeking.

### Objective

However, to our knowledge, dietary and nutrition information seeking using various web-based and offline media sources has not yet been explored in the context of food literacy and diet quality. Taken together, the above findings indicate that very little is known about people’s information-seeking behavior toward healthy eating and its potential consequences [47]. For potential correlates of dietary and nutrition information seeking, this study particularly focused on health literacy [48], food literacy [49], and diet quality [50-52], all of which were assessed using validated tools, as well as important sociodemographic variables (sex, age, and education level) [12,22,26,28,29,33,36,37,53,54]. To achieve sufficient distribution of health literacy, food literacy, and diet quality within the data set, our population consisted of not only the general public but also health professionals allied to nutrition (eg, dietitians, registered dietitians, physicians, and dentists). Thus, the aim of this cross-sectional study was to examine the prevalence and correlates of dietary and nutrition information seeking from various web-based and offline media sources among Japanese adults aged 20 to 79 years.

## Methods

### Study Procedure and Participants

**Figure 1:**
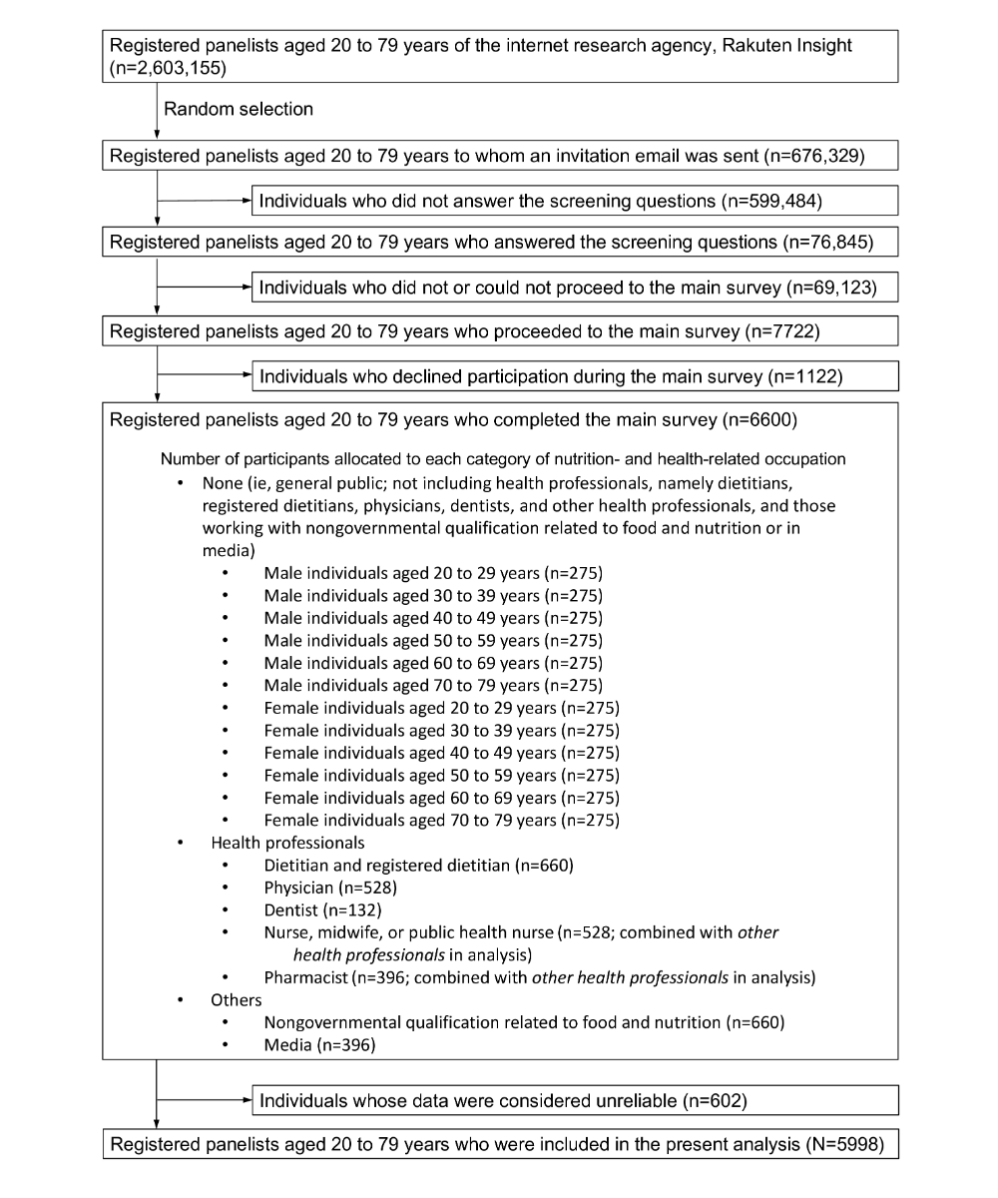
Flow diagram of the participants included in the analysis.

This cross-sectional study was based on data obtained from a web-based questionnaire survey. This paper was prepared in accordance with the CHERRIES (Checklist for Reporting Results of Internet E-Surveys) [55], where applicable. Figure 1 shows a flow diagram of participant selection. The target sample consisted of 6600 Japanese adults aged 20 to 79 years, including not only the general public but also health professionals allied to nutrition (eg, dietitians, registered dietitians, physicians, and dentists). Exclusion criteria were age outside the range of 20 to 79 years and working as an unrelated health professional (eg, veterinarians, dental hygienists, assistant nurses, clinical psychologists, and nurse practitioners).

Data collection was conducted by an internet research agency, Rakuten Insight [56]. With 2.2 million monitors nationwide, Rakuten Insight monitors fraudulent registrants, such as impersonators and duplicate registrants, by sharing the basic registration information of members. Furthermore, as part of the quality control of survey results, Rakuten Insight implemented an automated computerized checking system. Participants were recruited from among the registered panelists of the research agency. An email with an invitation to participate and a web page link to the survey was sent to a randomized list (n=676,329, 26.1%) of registered panelists aged 20 to 79 years (n=2,603,155; unfortunately, information on the sociodemographic characteristics of all the registered panelists in Rakuten Insight is not available [56]). Initially, a study summary was provided, and only individuals who agreed to participate could proceed to the screening stage (76,845/676,329, 11.36%). As recruitment was stratified by age, sex, and occupation, participants could proceed to the main survey only if there was a vacancy in the relevant category (eg, male individuals aged 20 to 79 years; Figure 1). As a result, among the 76,845 individuals in the screening stage, 7722 (10.05%) proceeded to the main survey, of whom 1122 (14.53%) did not complete all questions. Data collection started on February 10, 2023, and ended on March 16, 2023, when quotas for the main survey were filled (n=6600). The sample size was determined primarily based on feasibility and financial limitations. For analysis, we excluded individuals whose answers were considered unreliable based on their answer to the following question: “This question is for the purpose of investigating ‘misplaced’ answers when responding to the survey. Please select *neither agree or disagree* from the following options.” As a result, of the 6600 individuals who completed the survey, 314 (4.76%) individuals whose responses were *strongly agree*, *agree*, *disagree*, or *strongly disagree* were excluded. Further, we excluded individuals whose data were considered unreliable with regard to body height (≥200 cm; 2/6600, 0.03%) or energy intake (<800 or >4200 kcal/day for male individuals and <500 or >3500 kcal/day for female individuals [57]; 286/6600, 4.33%). Consequently, the final analysis sample comprised 5998 individuals (male individuals: n=2687, 44.8%; female individuals: n=3311, 55.2%) aged 20 to 79 years. The respondents included in the present analysis (N=5998) differed somewhat from those excluded from the analysis (n=602). The excluded respondents were more likely to be male and have higher means of age and BMI, lower household income, and nongovernmental qualifications related to food and nutrition.

### Ethical Considerations

The study was conducted according to the guidelines of the Declaration of Helsinki, and all procedures involving human participants were approved by the Ethics Committee of the University of Tokyo Faculty of Medicine (protocol code: 2022288NI; date of approval: January 13, 2023). Informed consent was obtained on the website from all individuals involved in the study. The participants were compensated with standard Rakuten Insight incentives (points redeemable for cash or merchandise) for completing the survey.

### Assessment of Sociodemographic Characteristics

All the questions used in this study were prepared by the first, second, and third authors. Sex (assigned at birth) was self-selected as either male or female. Age (in years) was also self-reported. Body weight and height were self-reported and used to calculate BMI (in kg/m^2^), with 3 weight status categories: underweight (<18.5), normal weight (≥18.5 to <25), and overweight (≥25) [58]. The following variables were also used in this study (categorization shown in parentheses): education level (junior high or high school, junior college or technical school, university or higher, and others), household income (<4 million Japanese yen, 4 to 7 million Japanese yen, >7 million Japanese yen, and unknown or do not want to answer; US $1=JPY 148.22), employment status (none, student, part-time job, and full-time job), marital status (unmarried, married, and do not want to answer), living alone (no and yes), presence of chronic diseases (eg, hypertension, hyperlipidemia, and diabetes; no and yes), and smoking status (never, past, and current). In addition, based on the reported postal code of the home address, each participant was grouped into 1 of 6 regions (Hokkaido and Tohoku, Kanto, Hokuriku and Tokai, Kinki, Chugoku and Shikoku, and Kyushu). Participants were also grouped into 1 of 3 municipality levels (ward, city, and town and village). The category *missing* was created for those whose reported postal codes were incomplete for the municipality level variable. Further, participants were categorized according to nutrition and health-related occupations (Figure 1): none (ie, the general public), nongovernmental qualification related to food and nutrition, media, dietitian and registered dietitian, physician and dentist, and other health professional (ie, nurse, midwife, public health nurse, and pharmacist).

### Assessment of Health Literacy

Health literacy was assessed using the Communicative and Critical Health Literacy scale, which was developed and validated for the Japanese population [48]. On the basis of a 5-point Likert scale (1 = *strongly disagree*; 5 = *strongly agree*), each participant was asked whether they were able to (1) collect health-related information from various sources, such as newspapers, books, television, and the internet; (2) extract the information they wanted; (3) understand and communicate the obtained information; (4) consider the credibility of the information; and (5) make decisions based on the information, specifically in the context of health-related issues. The health literacy score was calculated as the average of all items, meaning that the higher the score, the higher the health literacy (with possible scores ranging from 1 to 5) [48].

### Assessment of Food Literacy

As described in detail in Multimedia Appendix 1 [49], food literacy was assessed using the Japanese version of the 29-item Dutch Self-Perceived Food Literacy scale [49]. This is an expert-based, theory-driven, and validated tool for measuring food literacy with respect to healthy eating and focuses on 8 domains: food preparation skills (6 items), resilience and resistance (6 items), healthy snack styles (4 items), social and conscious eating (3 items), examining food labels (2 items), daily food planning (2 items), healthy budgeting (2 items), and healthy food stockpiling (4 items) [49]. Participants were asked to answer all the questions based on a 5-point Likert scale (1=*not at all* or *never*; 5=*yes* or *always*). The food literacy score was calculated as the average of all items, with negative items reversed, indicating that the higher the score, the higher the food literacy (with possible scores ranging from 1 to 5) [49].

### Assessment of Diet Quality

As a measure of diet quality, the Healthy Eating Index (HEI)-2020 [50] was used. The HEI-2020 is an established, 100-point scale to assess compliance with the 2020 to 2025 Dietary Guidelines for Americans [2], with a higher score indicating a better quality of overall diet. The HEI-2020 consists of 9 adequacy components (eg, fruits, vegetables, and whole grains) and 4 moderation components (eg, sodium and added sugars). The efficacy of the HEI-2015, which completely aligns with the HEI-2020 [50], in assessing the overall diet quality of Japanese people has been supported by our previous analyses [59,60]. Dietary intake information was collected using a validated, short version of the Meal-based Diet History Questionnaire [51,52,61]. A detailed description of the diet quality assessment is presented in Multimedia Appendix 2 [2,50-52,59-63].

### Assessment of Dietary and Nutrition Information Seeking Through Various Media Sources

The assessment of dietary and nutrition information seeking through various web-based and offline media sources was conducted using a series of 2 questions, as informed by previous studies [11,25,27,28,30,35-38,64]. Participants were first asked the following question: “Which of the following sources do you routinely use as a source of information about diet and nutrition? Please select all that apply (as many as you want)*.*” The list of sources of dietary and nutrition information shown was as follows: (1) television, (2) radio, (3) newspapers, (4) books and magazines, (5) municipal newsletters, (6) websites of government and medical manufacturers, (7) web searches, (8) news applications, (9) video sites (eg, YouTube [Google LLC]), and (10) social networking sites (eg, Twitter [Twitter Inc], Instagram [Meta Platforms Inc], and Facebook [Meta Platforms Inc]); 3 other face-to-face information sources (ie, hospitals and drug stores, family, and friends) were also shown, which are beyond the scope of this analysis. These were based on items used in a national survey conducted by the Ministry of Internal Affairs and Communications [65]. Participants were then asked the second question: “Do you think that dietary and nutrition information obtained from the sources listed below is reliable?” The list of dietary and nutrition information sources displayed was customized for each participant and contained only the sources the participant chose in the first question. For each of the dietary and nutrition information sources, a 5-point Likert scale category was provided (*strongly disagree*, *agree*, *neither agree or disagree*, *agree*, and *strongly agree*). Sources used to seek dietary and nutrition information were defined based on participants’ responses (*agree* or *strongly agree*) regarding the reliability of the sources.

### Statistical Analysis

All statistical analyses were performed using the SAS statistical software (version 9.4; SAS Institute Inc). Descriptive data are presented as frequencies and percentages of participants for categorical variables and means and SDs for continuous variables. Associations among the top media sources (defined a priori as >10% prevalence) used for seeking dietary and nutrition information were examined using a chi-square test. Associations of dietary and nutrition information seeking through the top media sources with participant characteristics were also examined using a chi-square test. Finally, multivariable odds ratios and their 95% CIs for dietary and nutrition information seeking through each media source according to participant characteristics were calculated using multiple logistic regression. For each media source, a model was constructed, which included dietary and nutrition information seeking through the media source as the dependent variable and sex (reference: male), age (continuous), weight status (reference: normal weight), education level (reference: junior high or high school), household income (reference: <4 million Japanese yen; US $1=JPY 148.22), employment status (reference: none), marital status (reference: unmarried), living alone (reference: no), presence of chronic disease (reference: no), smoking status (reference: never), region (reference: Kanto), municipality level (reference: ward), nutrition- and health-related occupations (reference: none, ie, general public), health literacy score (continuous), food literacy score (continuous), HEI-2020 score (continuous), and dietary and nutrition information seeking through 5 other media sources (reference: no for each) as the independent variables. The analysis was repeated separately for the general public and health professionals allied to nutrition. We considered 2-tailed *P* values <.05 statistically significant. We decided to treat age, health literacy score, food literacy score, and HEI-2020 score as continuous variables in our final analyses after observing similar findings when these variables were treated as categorical variables (20 to 39, 40 to 59, and 60 to 79 years for age and quartiles for others; data not shown).

## Results

### Basic Characteristics of Study Participants

This analysis included 5998 individuals (male individuals: n=2687, 44.8%; female individuals: n=3311, 55.2%) aged 20 to 79 years (Table 1).

**Table 1:** Basic characteristics of the study participants (N=5998).

			Values
**Sex, n (%)**			
	Male	2687 (44.8)	
	Female	3311 (55.2)	
Age (y), mean (SD)			46.8 (15.1)
**Weight status^a^, n (%)**			
	Underweight	783 (13.05)	
	Normal weight	4171 (69.54)	
	Overweight	1044 (17.41)	
**Education level, n (%)**			
	Junior high or high school	951 (15.86)	
	Junior college or technical school	1394 (23.24)	
	University or higher	3623 (60.4)	
	Other	30 (0.5)	
**Household income^b^, n (%)**			
	<4 million Japanese yen	1130 (18.84)	
	4 to 7 million Japanese yen	1596 (26.61)	
	>7 million Japanese yen	2297 (38.3)	
	Unknown or do not want to answer	975 (16.26)	
**Employment status, n (%)**			
	None	1010 (16.84)	
	Student	87 (1.45)	
	Part-time job	1025 (17.09)	
	Full-time job	3876 (64.62)	
**Marital status, n (%)**			
	Unmarried	2305 (38.43)	
	Married	3629 (60.5)	
	Do not want to answer	64 (1.07)	
**Living alone, n (%)**			
	No	4682 (78.06)	
	Yes	1316 (21.94)	
**Presence of chronic disease, n (%)**			
	No	3849 (64.17)	
	Yes	2149 (35.83)	
**Smoking status, n (%)**			
	Never	3886 (64.79)	
	Past	1213 (20.22)	
	Current	899 (14.99)	
**Region, n (%)**			
	Hokkaido and Tohoku	608 (10.14)	
	Kanto	2377 (39.63)	
	Tokai and Hokuriku	901 (15.02)	
	Kinki	1072 (17.87)	
	Chugoku and Shikoku	475 (7.92)	
	Kyushu	565 (9.42)	
**Municipality level, n (%)**			
	Ward	2300 (38.35)	
	City	3267 (54.47)	
	Town and village	321 (5.35)	
	Missing	110 (1.83)	
**Nutrition- and health-related occupations, n (%)**			
	None (ie, general public)	3021 (50.37)	
	Nongovernmental qualification related to food and nutrition	504 (8.4)	
	Media	359 (5.99)	
	Dietitian and registered dietitian	631 (10.52)	
	Physician and dentist	602 (10.04)	
	Other health professional	881 (14.69)	
Health literacy score (score range 1 to 5), mean (SD)			3.55 (0.60)
Food literacy score (score range 1 to 5), mean (SD)			3.18 (0.43)
Healthy Eating Index-2020 (score range 0 to 100), mean (SD)			50.4 (7.5)

^a^Underweight, normal weight, and overweight were defined as BMIs of <18.5, ≥18.5 to <25, and ≥25 kg/m^2^, respectively.

^b^US $1=JPY 148.22.

The mean BMI was 22.1 (SD 3.6) kg/m^2^; 69.54% (4171/5998) of the participants were categorized as having normal weight. Most participants had a high educational background (university or higher: 3623/5998, 60.4%) and full-time jobs (3876/5998, 64.62%) and were never (3886/5998, 64.79%) or past (1213/5998, 20.22%) smokers. Owing to the sampling design, approximately half (3021/5998, 50.37%) of the participants were members of the general public, whereas the remainder were in nutrition- and health-related occupations, such as dietitians and registered dietitians (631/5998, 10.52%) and physicians and dentists (602/5998, 10.04%).

When participants were allowed to choose with multiple options, the prevalence of diet and nutrition information seeking through various media sources among Japanese adults was as follows: 32.89% (1973/5998) for television, 5.3% (318/5998) for radio, 15.02% (901/5998) for newspapers, 11.62% (697/5998) for books and magazines, 3.52% (211/5998) for municipal newsletters, 16.62% (997/5998) for websites of government and medical manufacturers, 22.22% (1333/5998) for web searches, 9.85% (591/5998) for news applications, 10.57% (634/5998) for video sites (eg, YouTube), and 8.65% (519/5998) for social networking sites (eg, Twitter, Instagram, and Facebook). Thus, the major sources of information (>10% of prevalence) were television (1973/5998, 32.89%), web searches (1333/5998, 22.22%), websites of government and medical manufacturers (997/5998, 16.62%), newspapers (901/5998, 15.02%), books and magazines (697/5998, 11.62%), and video sites (eg, YouTube; 634/5998, 10.57%). These were then examined in subsequent analyses as the top media sources. Dietary and nutrition information seeking through these top 6 media sources was significantly associated with each other, except for a null association between dietary and nutrition information seeking through newspapers and dietary and nutrition information seeking through video sites (Multimedia Appendix 3). Dietary and nutrition information seeking via the top 6 media sources differed significantly according to participant characteristics, with a few exceptions, such as weight status, region, and municipality level (Multimedia Appendix 4).

### Associations of Participant Characteristics With Diet and Nutrition Information Seeking Through the Top 6 Media Sources

Multivariable odds ratios (with their 95% CIs) for dietary and nutrition information seeking through each media source are shown in Table 2 (for television, web searches, and websites of government and medical manufacturers) and Table 3 (for newspapers, books and magazines, and video sites).

**Table 2:** Associations of participant characteristics with diet and nutrition information seeking through the top 3 media sources (ie, television, web searches, and websites of government and medical manufacturers) among Japanese adults (N=5998)^a^.

			Television, OR^b^ (95% CI)^c^		Web searches, OR (95% CI)^d^		Websites of government and medical manufacturers, OR (95% CI)^e^
Female sex (reference: male)			*1.68 (1.44-1.97)*		0.95 (0.80-1.14)		*0.78 (0.64-0.95)*
Age (per 1-year increment)			1.00 (0.996-1.01)		1.00 (0.998-1.01)		*0.987 (0.980-0.994)*
**Weight status (reference: normal weight)^f^**							
	Underweight	0.94 (0.78-1.13)		*1.31 (1.07-1.60)*		1.07 (0.86-1.34)	
	Overweight	0.98 (0.83-1.16)		0.93 (0.77-1.12)		1.07 (0.87-1.33)	
**Education level (reference: junior high or high school)**							
	Junior college or technical school	0.84 (0.69-1.03)		1.08 (0.86-1.36)		1.12 (0.84-1.50)	
	University or higher	*0.77 (0.64-0.93)*		0.89 (0.72-1.09)		*1.41 (1.08-1.83)*	
	Other	0.36 (0.13-1.01)		0.48 (0.14-1.71)		1.37 (0.41-4.61)	
**Household income (reference: <4 million JPY)^g^**							
	4 to 7 million JPY	1.01 (0.84-1.22)		1.06 (0.86-1.31)		1.07 (0.84-1.37)	
	>7 million JPY	1.10 (0.90-1.34)		0.87 (0.69-1.09)		1.12 (0.86-1.44)	
	Unknown or do not want to answer	0.90 (0.72-1.12)		0.80 (0.62-1.02)		0.95 (0.71-1.26)	
**Employment status (reference: none)**							
	Student	1.41 (0.82-2.43)		*0.39 (0.19-0.81)*		1.33 (0.69-2.55)	
	Part-time job	1.14 (0.92-1.41)		1.07 (0.84-1.37)		0.75 (0.56-1.01)	
	Full-time job	1.02 (0.83-1.26)		1.02 (0.81-1.30)		1.05 (0.80-1.39)	
**Marital status (reference: unmarried)**							
	Married	*1.23 (1.04-1.44)*		1.07 (0.89-1.29)		0.83 (0.68-1.01)	
	Do not want to answer	0.86 (0.44-1.69)		0.50 (0.21-1.20)		0.33 (0.11-1.02)	
Living alone (reference: no)			*0.80 (0.66-0.97)*		1.17 (0.95-1.44)		0.96 (0.76-1.20)
Presence of chronic disease (reference: no)			0.91 (0.79-1.04)		1.00 (0.86-1.16)		*1.30 (1.10-1.54)*
**Smoking status (reference: never)**							
	Past	0.87 (0.73-1.02)		1.05 (0.88-1.26)		1.01 (0.83-1.24)	
	Current	0.93 (0.77-1.12)		1.03 (0.84-1.26)		0.84 (0.66-1.06)	
**Region (reference: Kanto)**							
	Hokkaido and Tohoku	1.01 (0.81-1.25)		0.87 (0.69-1.11)		0.94 (0.72-1.22)	
	Tokai and Hokuriku	1.12 (0.93-1.35)		1.00 (0.82-1.23)		0.88 (0.70-1.10)	
	Kinki	1.06 (0.89-1.26)		0.92 (0.76-1.12)		0.96 (0.77-1.18)	
	Chugoku and Shikoku	1.18 (0.93-1.50)		0.89 (0.68-1.16)		0.91 (0.68-1.22)	
	Kyushu	*1.27 (1.02-1.58)*		*0.76 (0.59- 0.98)*		0.89 (0.67-1.16)	
**Municipality level (reference: ward)**							
	City	0.91 (0.80-1.03)		1.11 (0.96-1.28)		1.02 (0.87-1.20)	
	Town and village	0.91 (0.69-1.21)		0.82 (0.59-1.14)		1.22 (0.87-1.71)	
	Missing	0.89 (0.55-1.43)		0.94 (0.54-1.64)		0.97 (0.53-1.75)	
**Nutrition- and health-related occupations (reference: none, ie, general public)**							
	Nongovernmental qualification related to food and nutrition	*0.61 (0.48-0.79)*		0.87 (0.66-1.14)		1.02 (0.76-1.37)	
	Media	1.18 (0.91-1.54)		0.80 (0.59-1.10)		0.76 (0.53-1.09)	
	Dietitian and registered dietitian	*0.57 (0.44-0.72)*		0.88 (0.67-1.14)		*2.60 (2.00-3.37)*	
	Physician and dentist	*0.55 (0.43-0.71)*		1.17 (0.90-1.52)		1.29 (0.97-1.71)	
	Other health professional	0.89 (0.74-1.09)		0.92 (0.74-1.16)		*1.43 (1.12-1.83)*	
Health literacy score (per 1-point increment)			*1.40 (1.25-1.57)*		*1.60 (1.40-1.82)*		*1.81 (1.57-2.09)*
Food literacy score (per 1-point increment)			*0.65 (0.55-0.77)*		0.85 (0.70-1.02)		*1.98 (1.62-2.44)*
Healthy Eating Index-2020 (per 1-point increment)			1.00 (0.993-1.01)		1.00 (0.994-1.01)		1.01 (0.999-1.02)

^a^Odds ratio (OR) for diet and nutrition information seeking through each media source in comparison with the reference category of each variable. Statistically significant values are italicized (*P*<.05).

^b^OR: odds ratio.

^c^Model with diet and nutrition information seeking through television as the dependent variable and variables listed in the first column and diet and nutrition information seeking through 5 other media sources (web searches, websites of government and medical manufacturers, newspapers, books and magazines, and video sites; no or yes for each) as the independent variables.

^d^Model with diet and nutrition information seeking through web searches as the dependent variable and variables listed in the first column and diet and nutrition information seeking through 5 other media sources (television, websites of government and medical manufacturers, newspapers, books and magazines, and video sites; no or yes for each) as the independent variables.

^e^Model with diet and nutrition information seeking through websites of government and medical manufacturers as the dependent variable and variables listed in the first column and diet and nutrition information seeking through 5 other media sources (television, web searches, newspapers, books and magazines, and video sites; no or yes for each) as the independent variables.

^f^Underweight, normal weight, and overweight were defined having BMIs of <18.5, ≥18.5 to <25, and ≥25 kg/m^2^, respectively.

^g^US $1=JPY 148.22.

**Table 3:** Associations of participant characteristics with diet and nutrition information seeking through the top 4th to 6th media sources (ie, newspapers, books and magazines, and video sites) among Japanese adults (N=5998)^a^.

		Newspapers, OR^b^ (95% CI)^c^	Books and magazines, OR (95% CI)^d^	Video sites (eg, YouTube), OR (95% CI)^e^
Female sex (reference: male)		*0.69 (0.56-0.86)*	*1.26 (1.001-1.59)*	*0.66 (0.53-0.83)*
Age (per 1-year increment)		*1.06 (1.05-1.07)*	1.00 (0.99-1.01)	*0.97 (0.96-0.98)*
**Weight status (reference: normal weight)^f^**				
	Underweight	1.29 (0.998-1.66)	0.79 (0.61-1.03)	0.84 (0.64-1.11)
	Overweight	*0.70 (0.56-0.88)*	*1.31 (1.02-1.67)*	0.90 (0.69-1.15)
**Education level (reference: junior high or high school)**				
	Junior college or technical school	0.95 (0.72-1.26)	1.13 (0.82-1.55)	0.96 (0.72-1.27)
	University or higher	*1.45 (1.14-1.86)*	1.17 (0.87-1.57)	*0.67 (0.52-0.88)*
	Other	2.10 (0.69-6.41)	1.42 (0.40-5.06)	1.23 (0.35-4.28)
**Household income (reference: <4 million JPY)^g^**				
	4 to 7 million JPY	1.01 (0.78-1.31)	1.11 (0.84-1.46)	1.03 (0.78-1.36)
	>7 million JPY	0.98 (0.74-1.29)	1.15 (0.85-1.53)	1.12 (0.83-1.51)
	Unknown or do not want to answer	0.85 (0.63-1.14)	0.85 (0.61-1.18)	0.82 (0.58-1.15)
**Employment status (reference: none)**				
	Student	1.32 (0.50-3.48)	2.07 (0.99-4.31)	0.92 (0.47-1.79)
	Part-time job	1.25 (0.95-1.66)	1.10 (0.79-1.52)	0.84 (0.60-1.16)
	Full-time job	1.26 (0.95-1.66)	1.15 (0.84-1.59)	0.75 (0.55-1.02)
**Marital status (reference: unmarried)**				
	Married	1.04 (0.82-1.31)	0.86 (0.68-1.09)	0.82 (0.64-1.03)
	Do not want to answer	0.67 (0.23-1.98)	0.95 (0.37-2.44)	1.35 (0.56-3.25)
Living alone (reference: no)		*0.51 (0.38-0.68)*	1.12 (0.86-1.46)	1.15 (0.88-1.50)
Presence of chronic disease (reference: no)		0.96 (0.80-1.15)	1.03 (0.85-1.25)	1.03 (0.84-1.27)
**Smoking status (reference: never)**				
	Past	0.88 (0.71-1.09)	1.02 (0.81-1.30)	*1.44 (1.14-1.83)*
	Current	0.79 (0.61-1.02)	*0.70 (0.52-0.95)*	*1.33 (1.03-1.73)*
**Region (reference: Kanto)**				
	Hokkaido and Tohoku	*1.47 (1.11-1.96)*	1.09 (0.81-1.47)	1.27 (0.94-1.72)
	Tokai and Hokuriku	1.27 (0.98-1.63)	1.07 (0.82-1.39)	1.06 (0.81-1.40)
	Kinki	1.16 (0.91-1.47)	0.94 (0.74-1.21)	0.93 (0.71-1.21)
	Chugoku and Shikoku	*1.74 (1.28-2.37)*	0.96 (0.68-1.35)	1.19 (0.84-1.68)
	Kyushu	*1.41 (1.05-1.90)*	0.84 (0.61-1.17)	1.36 (0.996-1.86)
**Municipality level (reference: ward)**				
	City	1.07 (0.90-1.28)	0.90 (0.75-1.08)	1.00 (0.82-1.21)
	Town and village	1.32 (0.92-1.89)	0.84 (0.56-1.25)	1.15 (0.77-1.72)
	Missing	0.80 (0.39-1.64)	1.02 (0.53-1.95)	1.14 (0.60-2.14)
**Nutrition- and health-related occupations (reference: none, ie, general public)**				
	Nongovernmental qualification related to food and nutrition	1.21 (0.87-1.69)	*1.64 (1.20-2.24)*	1.23 (0.91-1.66)
	Media	*1.49 (1.07-2.07)*	1.35 (0.94-1.96)	*0.51 (0.32-0.81)*
	Dietitian and registered dietitian	0.79 (0.55-1.12)	*1.97 (1.46-2.64)*	*0.54 (0.37-0.79)*
	Physician and dentist	*0.63 (0.46-0.87)*	1.03 (0.72-1.46)	1.03 (0.72-1.46)
	Other health professional	*0.64 (0.47-0.88)*	1.04 (0.77-1.41)	*0.59 (0.43-0.81)*
Health literacy score (per 1-point increment)		*1.27 (1.09-1.49)*	*1.48 (1.25-1.75)*	*1.33 (1.13-1.56)*
Food literacy score (per 1-point increment)		1.14 (0.91-1.43)	*2.09 (1.64-2.66)*	*1.53 (1.19-1.96)*
Healthy Eating Index-2020 (per 1-point increment)		*1.02 (1.01-1.03)*	*1.03 (1.02-1.04)*	1.01 (0.99-1.02)

^a^Odds ratio (OR) for diet and nutrition information seeking through each media source in comparison with the reference category of each variable. Statistically significant values are italicized (*P*<.05).

^b^OR: odds ratio.

^c^Model with diet and nutrition information seeking through newspapers as the dependent variable and variables listed in the first column and diet and nutrition information seeking through 5 other media sources (television, web searches, websites of government and medical manufacturers, books and magazines, and video sites; no or yes for each) as the independent variables.

^d^Model with diet and nutrition information seeking through books and magazines as the dependent variable and variables listed in the first column and diet and nutrition information seeking through 5 other media sources (television, web searches, websites of government and medical manufacturers, newspapers, and video sites; no or yes for each) as the independent variables.

^e^Model with diet and nutrition information seeking through video sites (eg, YouTube) as the dependent variable and variables listed in the first column and diet and nutrition information seeking through 5 other media sources (television, web searches, websites of government and medical manufacturers, newspapers, and books and magazines; no or yes for each) as the independent variables.

^f^Underweight, normal weight, and overweight were defined having BMIs of <18.5, ≥18.5 to <25, and ≥25 kg/m^2^, respectively.

^g^US $1=JPY 148.22.

Information seeking through watching television was associated with female sex, lower education, being married, living with someone, living in the Kyushu region (compared with living in the Kanto region), belonging to the general public (compared with having a nongovernmental qualification related to food and nutrition, being a dietitian or registered dietitian, and being a physician or dentist), a higher score of health literacy, and a lower score of food literacy. Web searches were associated with underweight (compared with normal weight), nonemployment status (compared with students), living in the Kanto region (compared with living in the Kyushu region), and a higher score of health literacy. Use of websites of government and medical manufacturers was associated with male sex; younger age; higher education; the presence of chronic disease; being a dietitian or registered dietitian, physician or dentist, or other health professional (compared with belonging to the general public); and higher scores for health literacy and food literacy. Seeking information from newspapers was associated with male sex; older age; higher education; living with someone; living in the Hokkaido and Tohoku, Chugoku and Shikoku, or Kyushu regions; working in the media (compared with belonging to the general public); and higher scores for health literacy and the HEI-2020. In addition, overweight and being a physician or dentist or other health professional (compared with belonging to the general public) were less likely to seek information from newspapers. Information seeking through books and magazines was associated with female sex; overweight; never smoking (compared with current smoking); working with nongovernmental qualifications related to food and nutrition or as a dietitian or registered dietitian (compared with belonging to the general public); and higher scores for health literacy, food literacy, and the HEI-2020. The use of video sites was associated with male sex, younger age, lower education, past and current smoking, belonging to the general public (compared with working in the media, as a dietitian or registered dietitian, or as other health professional), and higher scores for health literacy and food literacy. The analysis was repeated separately for the general public (Multimedia Appendix 5) and health professionals allied to nutrition (Multimedia Appendix 6). In general, similar findings were observed in approximately 90% (81/90) of the associations examined, although some did not reach statistical significance.

## Discussion

### Principal Findings

To our knowledge, this is the first study to comprehensively examine the prevalence and correlates of dietary and nutrition information seeking from web-based and offline media sources. In this cross-sectional study of 5998 Japanese adults aged 20 to 79 years, the principal dietary and nutrition information platforms used by >10% of participants were television (1973/5998, 32.89%), web searches (1333/5998, 22.22%), websites of government and medical manufacturers (997/5998, 16.62%), newspapers (901/5998, 15.02%), books and magazines (697/5998, 11.62%), and video sites (eg, YouTube; 634/5998, 10.57%). After adjustment for potential confounding factors, health literacy was positively associated with the use of each of these sources. By contrast, food literacy showed an inverse association with watching television, whereas it was positively associated with the use of websites of government and medical manufacturers, books and magazines, and video sites. In addition, the media sources that were positively associated with diet quality were newspapers and books and magazines. Regarding sociodemographic variables, being female was associated with watching television and reading books and magazines, whereas being male was more likely to be associated with using websites of government and medical manufacturers, newspapers, and video sites. Older people were more likely to read newspapers for nutrition information, whereas younger people were more attracted to websites of government and medical manufacturers and video sites. People with higher education levels were more likely to use websites of government and medical manufacturers and newspapers but less likely to rely on television and video sites. Compared with the general public, dietitians and registered dietitians were more likely to use websites of government and medical manufacturers and books and magazines but less likely to use television and video sites.

### Comparison With Prior Work

To our knowledge, only a few studies have investigated the lifestyle (especially dietary) profiles of people seeking dietary and nutrition information from various media sources [25,26,35-40]. This type of study is of utmost importance, given not only that our eating habits have a significant impact on our health [1] but also that the general media landscape is filled with dietary misinformation [14-17]. In this study, after adjustment for potential confounding factors, nutrition information seeking from newspapers and books and magazines (but not television, web searches, websites of government and medical manufacturers, and video sites) was positively associated with diet quality. Thus, the association between nutrition information seeking and diet quality was only observed for offline media. This may be explained by the nature of offline media use, which permits the skimming, reading, and rereading of information, thereby encouraging an active learning process [26,35,38] that may lead to favorable dietary behaviors. Positive associations between the use of offline media and the consumption of fruits and vegetables have been consistently observed in previous studies [26,35,38], with the exception of 1 study [25]. Conversely, previous studies failed to find a significant association between the use of web-based media and fruit and vegetable consumption [25,26,36,37], with the exception of 1 study [35]. Null associations were also observed in this study despite our classification of internet use into several categories (ie, web searches, websites of government and medical manufacturers, video sites, news applications, and social networking sites). With regard to watching television, previous studies have generally not shown favorable associations with eating behaviors [25,26,35,38], which is consistent with the present findings. This fits well with the suggestion that television creates only a passive learning environment, which is unsuitable for the delivery of meaning, knowledge, or context [26,38].

In line with previous studies [19], we found that health literacy was positively associated with the use of all media sources for nutrition information seeking. A novel finding of our study was that food literacy was positively associated with nutrition information seeking using websites of government and medical manufacturers, books and magazines, and video sites. Although we are not aware of previous research on this topic, the findings are plausible, given that gathering information from these sources is an active process and is, therefore, more prevalent in health information–oriented, health-concerned individuals [38], including people with higher food literacy. Interestingly, we found an inverse association between nutrition information seeking using television and food literacy. The exact reason is unclear, but this might be attributable to the definition and components of the food literacy score measured in this study, which do not necessarily include information seeking skills but rather represent a collection of interrelated knowledge, skills, and behaviors needed for healthy eating [21]. For people with lower food literacy scores and who are unlikely to be interested in nutrition-related topics, television may be the easiest way to obtain dietary and nutrition information.

In this study, the top dietary and nutrition information source was television, followed by web searches, websites of government and medical manufacturers, newspapers, books and magazines, and video sites. This is consistent with a finding from a national survey conducted in 2019 that showed that a larger portion of the general Japanese population considers traditional media, such as television (52%), books and magazines (23%), and newspapers (23%), as information sources that influence their dietary habits, compared with the internet (17% for websites and 8% for social media; multiple choices allowed) [34]. This was particularly apparent in older adults [34]. This tendency to rely on traditional mass media (particularly television and newspapers) for seeking health information has been repeatedly observed in Asian populations, such as Japanese [30-34] and Hong Kong Chinese populations [22,54]. The reason is unknown, but it may be because Asian cultures often prioritize values of obedience, duty, and in-group harmony [66,67].

A unique characteristic of this study is its inclusion of health professionals allied to nutrition, such as dietitians and registered dietitians. This design allowed us to describe interesting pictures of and build knowledge on how the general public and health professionals related to nutrition use and benefit from dietary and nutrition information seeking differently. As expected, we found that compared with the general public, dietitians and registered dietitians were more likely to refer to websites of government and medical manufacturers and books and magazines and less likely to use television or video sites for nutrition information. Taken together with our findings on food literacy and diet quality, this study does suggest that dietary and nutrition information obtained from websites of government and medical manufacturers and books and magazines would be relatively high in quality and usefulness, whereas information obtained from television (and video sites) would be relatively low in quality and useless. This interpretation is generally consistent with previous studies on the quality of nutrition-related information [13,68-74]. Nevertheless, no empirical evidence on the quality of web-based or offline dietary and nutrition information written in Japanese is available, and such investigations are warranted [17].

Studies have shown that being female and having a higher educational background are the strongest correlates of seeking health information from web-based sources [12,22,29,53,54]. Similar associations have also been suggested with regard to offline health information seeking [22,29,54], except for null or inverse associations between education and television exposure [38,75,76]. Studies also suggest that younger age is associated with web-based health information seeking [12,22,53], whereas older age is associated with offline health information seeking [22,28]. Our findings are generally consistent with these previous findings, except for findings regarding the role of sex. We found that being female was associated with the use of television and books and magazines, whereas being male was associated with the use of websites of government and medical manufacturers, newspapers, and video sites. The reason is unclear, but this may be an artifact of the self-selection process required to participate in the study. Alternatively, the use of websites of government and medical manufacturers, newspapers, and video sites in Japan may appear a male-dominated activity, which may be derived from the notion that such information-gathering skills have been historically stereotypically perceived as more masculine (eg, male individuals are good with technology and literacy) [77,78] or from a perception of lower degree of digital competence in women than in men [79].

In summary, although health-related information seeking varies across cultures and countries [23,80], all previous studies on nutrition information–seeking behaviors have been conducted in Western countries [11,25-28,35-38]. Therefore, our study of Japanese adults provides valuable insights into information-seeking behavior in relation to healthy eating and its potential consequences. This information may be used in future public health strategies aimed at improving health.

### Limitations

This study has several limitations. First, it was conducted using a cross-sectional design. Because the temporality of the associations is unknown, it is not possible to address causality or its direction. For example, we cannot determine whether obtaining information about diet and nutrition from books and magazines contributes to improved food literacy and diet quality, or vice versa. More prospective research is needed on this topic. Second, our sample was confined to individuals registered with an internet research agency and, therefore, limited to people with access to the internet, and only a small percentage of these individuals expressed interest in participating in the study. Thus, compared with a nationally representative population, there is likely to be a higher use of web-based media for gathering information on diet and nutrition and a lower use of offline media. In addition, by design, approximately half (2977/5998, 49.63%) of our participants were health professionals allied to nutrition. Consequently, the education level and household income of our participants were higher than those of a nationally representative sample (education: 54,760,800/100,247,800, 54.63% for junior high school or high school, 20,808,900/100,247,800, 20.76% for junior college or technical school, and 24,678,100/100,247,800, 24.62% for university or higher [81]; household income: 2943/6541, 45% for <4 million Japanese yen, 1760/6541, 26.9% for ≥4 to <7 million Japanese yen, and 1831/6541, 28% for ≥7 million Japanese yen [82]). Moreover, our participants appeared different from a nationally representative sample, at least in terms of mean (SD) height, weight, BMI, and HEI-2020 score (male individuals: 167.7, SD 6.9 cm; 67.4, SD 12.0 kg; 23.9, SD 3.6 kg/m^2^; and 51.3, SD 9.0, respectively; female individuals: 154.3, SD 6.7 cm; 53.6, SD 9.2 kg; 22.5, SD 3.7 kg/m^2^; and 52.9, SD 9.2, respectively) [34,59]. Taken together, although our sample size was large, the participants were not a nationally representative sample of the general Japanese population. For example, our study may have missed some socially susceptible groups (eg, people without homes or those receiving income support). By including these people, we may have found different correlates for seeking information about diet and nutrition from various web-based and offline media sources. Further research in a more representative sample is warranted.

Third, as with any survey based on a self-administered questionnaire, all the variables used in this study were based on self-report and may thus be subject to biased reporting despite our use of validated tools to assess health literacy [48], food literacy [49], and diet quality [50-52]. Regarding information-seeking behavior, several methodological studies have shown that self-reports of web-based activities [83], mobile phone use [84], and news exposure on television [85] often diverge from real behaviors. Thus, the self-reported responses regarding dietary and nutrition information seeking obtained in this study might be poor reflections of the truth. If so, the associations between media use for seeking dietary and nutrition information and the sociodemographic and lifestyle variables we observed may be generally diluted compared with the true associations. Therefore, the present findings should be interpreted with caution in this context, and efforts should be made to develop a validated scale to assess nutrition information–seeking behavior for use in future research. Fourth, as in many other studies [11,25-28,35-38,86], exposure to dietary and nutrition information sources was rather crudely measured in this study. The dichotomous nature of our measure did not permit a more refined measurement of variables, including an individual’s frequency of use (eg, yearly, weekly, and daily); the content, types, and quality of information participants were exposed to; and for whom the participants were seeking information. An in-depth analysis that incorporates these variables should be conducted to uncover the complexity of dietary and nutrition information–seeking behavior.

Fifth, although this study was conducted during a certain time window (February and March 2023), previous studies have suggested seasonal variations in dieting and weight loss attempts [87] and the frequency of internet searches for fitness and weight loss information [88]. Therefore, certain sociodemographic and lifestyle characteristics associated with media use for seeking dietary and nutrition information may manifest differently over time [86]. As such, it should be noted that this study provides only a snapshot in time. Future research should be designed to capture this dynamic nature of nutrition information–seeking behavior and its correlates. Finally, although we adjusted for various variables, we cannot rule out the possibility of residual confounding. Despite these limitations, the major strength of this study is its inclusion of a wide variety of dietary and nutrition information sources. Unlike a substantial majority of recent studies [11,23,36,37,86], our data allowed for differentiation among several web-based information sources (eg, web searches, websites of government and medical manufacturers, and video sites). This study also investigated more conventional, offline information sources (eg, television, newspapers, and books and magazines), which are still widely valued and used, in Asian countries at least [22,30-34,54]. People who use these information sources do not necessarily have the same approach and thus might not have the same profiles [89], which was clearly observed in this study.

### Practical Implications

Our findings have implications for public health. First, the study demonstrates that a wide variety of sources are regularly used for seeking dietary and nutrition information in Japan. However, it was clear that each source was preferred by users with distinct characteristics. On the basis of these differences in potential users, we speculated that each media source should have suitable topics and optimal information dissemination strategies. For example, it is ideal to use television, an entertainment-oriented medium, for constantly disseminating easy-to-understand messages for chronic disease prevention (eg, “make half your plate fruits & vegetables”) [2] to instigate health-related conversations with family and friends for people with lower levels of formal education [38,76]. This may be particularly relevant, considering the multitasking agenda of women, who are frequent users of television and who play key roles as health managers and family caregivers [79,90,91]. In addition, newspapers (as well as books and magazines) should perhaps include only a small number of high-quality articles on the importance of overall dietary patterns, a topic for which the most powerful evidence is accumulated in nutritional epidemiology [1,2,4]. These articles should be written by reputable journalists with adequate training on issues related to scientific methodology, food, and health [92]. If successful, newspapers may play a vital role in chronic disease prevention and management [12] among middle-aged and older individuals with a higher educational background [38,76]. Further, for every web-based media source, nutrition professionals are ultimately responsible for and able to contribute to the creation and promotion of evidence-based content on topics that are popular [16,17,93] among the public [13,94]. Simultaneously, consideration should be given to the practicality and utility of the information [6] as well as to the affective needs (eg, enjoyment) and cognitive needs (eg, interest) of information [15], all of which are essential to the effective dissemination of dietary and nutrition information.

Ultimately, however, it is the consumers who decide from what and where they learn and what they eat. Research shows that although consumers say that they primarily look for the source, the presence of a professional design, and a variety of other criteria when assessing the credibility of a website, internet users do not in practice check the “about us” sections of websites, try to find out who the authors or owners of sites are, or read disclaimers or disclosure statements [95]. As an initial step, the general public should be sufficiently informed that there is a huge amount of misinformation (and potentially disinformation, ie, false information that is intentional spread) about nutrition, particularly on the internet and social media [7]. More fundamentally, critical thinking, or the ability to objectively analyze facts to make decisions successfully [96], appears to be an essential skill in the present age of highly developed information [6,7]. Excellence in this type of thinking does not come naturally but must be systematically cultivated [7]. Therefore, systematic learning of critical thinking skills should be introduced in the compulsory education system, with a focus on understanding the scientific process while eliminating the sole focus on results, thereby empowering consumers to evaluate evidence and understand the evolving nature of science [6].

### Conclusions

This cross-sectional study identified various web-based and offline media sources regularly used by Japanese adults seeking nutrition information. Except for health literacy, each media source had unique correlates, suggesting large differences in potential users, relevant topics, and optimal information dissemination strategies among media sources. Perhaps the most concerning finding is the lack of positive associations between the use of 2 major sources (television and web searches) and food literacy and diet quality. By contrast, a promising finding is the positive associations of the use of websites of government and medical manufacturers, newspapers, books and magazines, and video sites with food literacy or diet quality. The present findings are a valuable scientific contribution to the development of effective promotional tactics and strategies for healthy eating.

## Appendix

Multimedia Appendix 1 Detailed description of the assessment of food literacy.

Multimedia Appendix 2 Detailed description of the assessment of diet quality.

Multimedia Appendix 3 Associations among the top 6 media sources used by Japanese adults for seeking diet and nutrition information (N=5998).

Multimedia Appendix 4 Prevalence of diet and nutrition information seeking through the top 6 media sources among Japanese adults, according to participant characteristics (N=5998).

Multimedia Appendix 5 Associations of participant characteristics with diet and nutrition information seeking through the top 6 media sources among Japanese adults belonging to the general public (3021/5998, 50.37%).

Multimedia Appendix 6 Associations of participant characteristics with diet and nutrition information seeking through the top 6 media sources among Japanese adults with nutrition and health-related occupations (2977/5998, 49.63%).

## Abbreviations

CHERRIES: Checklist for Reporting Results of Internet E-Surveys
HEI: Healthy Eating Index

## Data Availability

The data sets used or analyzed in this study are available from the corresponding author upon reasonable request.

## References

[1] GBD 2017 Diet Collaborators (2019). Health effects of dietary risks in 195 countries, 1990-2017: a systematic analysis for the Global Burden of Disease Study 2017. Lancet.

[2] Dietary guidelines for Americans, 2020-2025. 9th edition. U.S. Department of Agriculture and U.S. Department of Health and Human Services.

[3] Lane MM, Davis JA, Beattie S, Gómez-Donoso C, Loughman A, O'Neil A, Jacka F, Berk M, Page R, Marx W, Rocks T (2021). Ultraprocessed food and chronic noncommunicable diseases: a systematic review and meta-analysis of 43 observational studies. Obes Rev.

[4] Willett W, Rockström J, Loken B, Springmann M, Lang T, Vermeulen S, Garnett T, Tilman D, DeClerck F, Wood A, Jonell M, Clark M, Gordon LJ, Fanzo J, Hawkes C, Zurayk R, Rivera JA, De Vries W, Majele Sibanda L, Afshin A, Chaudhary A, Herrero M, Agustina R, Branca F, Lartey A, Fan S, Crona B, Fox E, Bignet V, Troell M, Lindahl T, Singh S, Cornell SE, Srinath Reddy K, Narain S, Nishtar S, Murray CJ (2019). Food in the Anthropocene: the EAT-Lancet Commission on healthy diets from sustainable food systems. Lancet.

[5] Schwingshackl L, Bogensberger B, Hoffmann G (2018). Diet quality as assessed by the healthy eating index, alternate healthy eating index, dietary approaches to stop hypertension score, and health outcomes: an updated systematic review and meta-analysis of cohort studies. J Acad Nutr Diet.

[6] Garza C, Stover PJ, Ohlhorst SD, Field MS, Steinbrook R, Rowe S, Woteki C, Campbell E (2019). Best practices in nutrition science to earn and keep the public's trust. Am J Clin Nutr.

[7] Diekman C, Ryan CD, Oliver TL (2023). Misinformation and disinformation in food science and nutrition: impact on practice. J Nutr.

[8] Dooley JA, Jones SC, Iverson D (2014). Using Web 2.0 for health promotion and social marketing efforts: lessons learned from Web 2.0 experts. Health Mark Q.

[9] Jenkins EL, Ilicic J, Barklamb AM, McCaffrey TA (2020). Assessing the credibility and authenticity of social media content for applications in health communication: scoping review. J Med Internet Res.

[10] Adamski M, Truby H, M Klassen K, Cowan S, Gibson S (2020). Using the internet: nutrition information-seeking behaviours of lay people enrolled in a massive online nutrition course. Nutrients.

[11] Pollard CM, Pulker CE, Meng X, Kerr DA, Scott JA (2015). Who uses the internet as a source of nutrition and dietary information? An Australian population perspective. J Med Internet Res.

[12] Mitsutake S, Takahashi Y, Otsuki A, Umezawa J, Yaguchi-Saito A, Saito J, Fujimori M, Shimazu T, INFORM Study Group (2023). Chronic diseases and sociodemographic characteristics associated with online health information seeking and using social networking sites: nationally representative cross-sectional survey in Japan. J Med Internet Res.

[13] Denniss E, Lindberg R, McNaughton SA (2023). Quality and accuracy of online nutrition-related information: a systematic review of content analysis studies. Public Health Nutr.

[14] Wang Y, McKee M, Torbica A, Stuckler D (2019). Systematic literature review on the spread of health-related misinformation on social media. Soc Sci Med.

[15] Vrinten J, Van Royen K, Pabian S, De Backer C, Matthys C (2022). Motivations for nutrition information-seeking behavior among Belgian adults: a qualitative study. BMC Public Health.

[16] Ramachandran D, Kite J, Vassallo AJ, Chau JY, Partridge SR, Freeman B, Gill T (2018). Food trends and popular nutrition advice online - implications for public health. Online J Public Health Inform.

[17] Murakami K, Shinozaki N, Kimoto N, Onodera H, Oono F, McCaffrey TA, Livingstone MB, Okuhara T, Matsumoto M, Katagiri R, Ota E, Chiba T, Nishida Y, Sasaki S (2023). Web-based content on diet and nutrition written in Japanese: infodemiology study based on Google trends and Google search. JMIR Form Res.

[18] Nutbeam D (1998). Health promotion glossary. Health Promot Int.

[19] Berkman ND, Sheridan SL, Donahue KE, Halpern DJ, Crotty K (2011). Low health literacy and health outcomes: an updated systematic review. Ann Intern Med.

[20] Spronk I, Kullen C, Burdon C, O'Connor H (2014). Relationship between nutrition knowledge and dietary intake. Br J Nutr.

[21] Vidgen HA, Gallegos D (2014). Defining food literacy and its components. Appetite.

[22] Wang MP, Viswanath K, Lam TH, Wang X, Chan SS (2013). Social determinants of health information seeking among Chinese adults in Hong Kong. PLoS One.

[23] Link E, Baumann E, Klimmt C (2021). Explaining online information seeking behaviors in people with different health statuses: German representative cross-sectional survey. J Med Internet Res.

[24] Case DO, Given LM (2007). Looking for Information: A Survey of Research on Information Seeking, Needs, and Behavior.

[25] Redmond N, Baer HJ, Clark CR, Lipsitz S, Hicks LS (2010). Sources of health information related to preventive health behaviors in a national study. Am J Prev Med.

[26] Beaudoin CE, Hong T (2011). Health information seeking, diet and physical activity: an empirical assessment by medium and critical demographics. Int J Med Inform.

[27] Goodman S, Hammond D, Pillo-Blocka F, Glanville T, Jenkins R (2011). Use of nutritional information in Canada: national trends between 2004 and 2008. J Nutr Educ Behav.

[28] Jacobs W, Amuta AO, Jeon KC (2017). Health information seeking in the digital age: an analysis of health information seeking behavior among US adults. Cogent Soc Sci.

[29] Hone T, Palladino R, Filippidis FT (2016). Association of searching for health-related information online with self-rated health in the European Union. Eur J Public Health.

[30] Inoue M, Shimoura K, Nagai-Tanima M, Aoyama T (2022). The relationship between information sources, health literacy, and COVID-19 knowledge in the COVID-19 infodemic: cross-sectional online study in Japan. J Med Internet Res.

[31] Otsuki A, Saito J, Yaguchi‐Saito A, Odawara M, Fujimori M, Hayakawa M, Katanoda K, Matsuda T, Matsuoka YJ, Takahashi H, Takahashi M, Inoue M, Yoshimi I, Kreps GL, Uchitomi Y, Shimazu T (2022). A nationally representative cross‐sectional survey on health information access for consumers in Japan: A protocol for the INFORM Study. World Med Health Policy.

[32] Takahashi Y, Ohura T, Ishizaki T, Okamoto S, Miki K, Naito M, Akamatsu R, Sugimori H, Yoshiike N, Miyaki K, Shimbo T, Nakayama T (2011). Internet use for health-related information via personal computers and cell phones in Japan: a cross-sectional population-based survey. J Med Internet Res.

[33] Ishikawa Y, Nishiuchi H, Hayashi H, Viswanath K (2012). Socioeconomic status and health communication inequalities in Japan: a nationwide cross-sectional survey. PLoS One.

[34] The national health and nutrition survey in Japan. Ministry of Health, Labour and Welfare, Japan.

[35] Freisling H, Haas K, Elmadfa I (2010). Mass media nutrition information sources and associations with fruit and vegetable consumption among adolescents. Public Health Nutr.

[36] Shahab L, Brown J, Gardner B, Smith SG (2014). Seeking health information and support online: does it differ as a function of engagement in risky health behaviors? Evidence from the health information national trends survey. J Med Internet Res.

[37] Gonzalez M, Sanders-Jackson A, Emory J (2016). Online health information-seeking behavior and confidence in filling out online forms among Latinos: a cross-sectional analysis of the California health interview survey, 2011-2012. J Med Internet Res.

[38] Dutta-Bergman MJ (2004). Primary sources of health information: comparisons in the domain of health attitudes, health cognitions, and health behaviors. Health Commun.

[39] Choi H, Jeong G (2021). Characteristics of the measurement tools for assessing health information-seeking behaviors in nationally representative surveys: systematic review. J Med Internet Res.

[40] Kim K, Shin S, Kim S, Lee E (2023). The relation between eHealth literacy and health-related behaviors: systematic review and meta-analysis. J Med Internet Res.

[41] Ioannidis JP (2013). Implausible results in human nutrition research. BMJ.

[42] Hébert JR, Hurley TG, Steck SE, Miller DR, Tabung FK, Peterson KE, Kushi LH, Frongillo EA (2014). Considering the value of dietary assessment data in informing nutrition-related health policy. Adv Nutr.

[43] Satija A, Yu E, Willett WC, Hu FB (2015). Understanding nutritional epidemiology and its role in policy. Adv Nutr.

[44] Waijers PM, Feskens EJ, Ocké MC (2007). A critical review of predefined diet quality scores. Br J Nutr.

[45] Baranowski T, Cullen KW, Nicklas T, Thompson D, Baranowski J (2003). Are current health behavioral change models helpful in guiding prevention of weight gain efforts?. Obes Res.

[46] Sexton-Dhamu MJ, Livingstone KM, Pendergast FJ, Worsley A, McNaughton SA (2021). Individual, social-environmental and physical-environmental correlates of diet quality in young adults aged 18-30 years. Appetite.

[47] Ruani MA, Reiss MJ, Kalea AZ (2023). Diet-nutrition information seeking, source trustworthiness, and eating behavior changes: an international web-based survey. Nutrients.

[48] Ishikawa H, Nomura K, Sato M, Yano E (2008). Developing a measure of communicative and critical health literacy: a pilot study of Japanese office workers. Health Promot Int.

[49] Poelman MP, Dijkstra SC, Sponselee H, Kamphuis CB, Battjes-Fries MC, Gillebaart M, Seidell JC (2018). Towards the measurement of food literacy with respect to healthy eating: the development and validation of the self perceived food literacy scale among an adult sample in the Netherlands. Int J Behav Nutr Phys Act.

[50] Shams-White MM, Pannucci TE, Lerman JL, Herrick KA, Zimmer M, Meyers Mathieu K, Stoody EE, Reedy J (2023). Healthy eating index-2020: review and update process to reflect the dietary guidelines for Americans,2020-2025. J Acad Nutr Diet.

[51] Murakami K, Shinozaki N, McCaffrey TA, Livingstone MB, Sasaki S (2021). Data-driven development of the meal-based diet history questionnaire for Japanese adults. Br J Nutr.

[52] Murakami K, Shinozaki N, Livingstone MB, Kimoto N, Masayasu S, Sasaki S (2022). Relative validity of the online meal-based diet history questionnaire for evaluating the overall diet quality and quality of each meal type in Japanese adults. Br J Nutr.

[53] Jia X, Pang Y, Liu LS (2021). Online health information seeking behavior: a systematic review. Healthcare (Basel).

[54] Guo N, Guo Z, Zhao S, Ho SY, Fong DY, Lai AY, Chan SS, Wang MP, Lam TH (2021). Digital inequalities in health information seeking behaviors and experiences in the age of web 2.0: a population-based study in Hong Kong. PLoS One.

[55] Eysenbach G (2004). Improving the quality of Web surveys: the Checklist for Reporting Results of Internet E-Surveys (CHERRIES). J Med Internet Res.

[56] Home page. Rakuten Insight.

[57] Bertoia ML, Rimm EB, Mukamal KJ, Hu FB, Willett WC, Cassidy A (2016). Dietary flavonoid intake and weight maintenance: three prospective cohorts of 124,086 US men and women followed for up to 24 years. BMJ.

[58] World Health Organization (2000). Obesity: preventing and managing the global epidemic. Report of a WHO consultation. World Health Organ Tech Rep Ser.

[59] Murakami K, Livingstone MB, Fujiwara A, Sasaki S (2020). Application of the healthy eating index-2015 and the nutrient-rich food index 9.3 for assessing overall diet quality in the Japanese context: different nutritional concerns from the US. PLoS One.

[60] Murakami K, Shinozaki N, Livingstone MB, Fujiwara A, Asakura K, Masayasu S, Sasaki S (2020). Meal and snack frequency in relation to diet quality in Japanese adults: a cross-sectional study using different definitions of meals and snacks. Br J Nutr.

[61] Murakami K, Shinozaki N, Kimoto N, Masayasu S, Sasaki S (2022). Relative validity of food intake in each meal type and overall food intake derived using the meal-based diet history questionnaire against the 4-day weighed dietary record in Japanese adults. Nutrients.

[62] Bowman SA, Clemens JC, Friday JE, Thoerig RC, Moshfegh A (2014). Food patterns equivalents database 2011-12: methodology and user guide. U.S. Department of Agriculture.

[63] (2015). Standard tables of food composition in Japan 2015 (7th revised edition). Council for Science and Technology, Ministry of Education, Culture, Sports, Science and Technology, Japan.

[64] Covolo L, Guana M, Bonaccorsi G, Brunelli L, Castaldi S, De Donno A, Mereu A, Verani M, Gelatti U (2022). Exploring the online health information-seeking behavior in a sample of Italian women: the "SEI Donna" study. Int J Environ Res Public Health.

[65] (2021). Information and communications in Japan. Ministry of Internal Affairs and Communications.

[66] Tata SP, Leong FT (1994). Individualism–collectivism, social-network orientation, and acculturation as predictors of attitudes toward seeking professional psychological help among Chinese Americans. J Couns Psychol.

[67] Shea M, Yeh C (2008). Asian American students' cultural values, stigma, and relational self-construal: correlates of attitudes toward professional help seeking. J Ment Health Couns.

[68] Sütcüoğlu O, Özay ZI, Özet A, Yazıcı O, Özdemir N (2023). Evaluation of scientific reliability and quality of YouTube videos on cancer and nutrition. Nutrition.

[69] Benajiba N, Alhomidi M, Alsunaid F, Alabdulkarim A, Dodge E, Chavarria EA, Aboul-Enein BH (2023). Video clips of the Mediterranean Diet on YouTube : a social media content analysis. Am J Health Promot.

[70] Lamb KL, Barker ME, Lynn A (2023). A content analysis of online videos containing dietary recommendations for gout and their alignment with evidence-based dietary guidelines. Public Health Nutr.

[71] Batar N, Kermen S, Sevdin S, Yıldız N, Güçlü D (2020). Assessment of the quality and reliability of information on nutrition after bariatric surgery on YouTube. Obes Surg.

[72] Long M, Forbes LE, Papagerakis P, Lieffers JR (2023). YouTube videos on nutrition and dental caries: content analysis. JMIR Infodemiology.

[73] Harrison K, Marske AL (2005). Nutritional content of foods advertised during the television programs children watch most. Am J Public Health.

[74] Story M, Faulkner P (1990). The prime time diet: a content analysis of eating behavior and food messages in television program content and commercials. Am J Public Health.

[75] De Jesus M, Xiao C (2012). Predicting internet use as a source of health information: a “Language Divide” among the Hispanic population in the United States. Policy Internet.

[76] Seo M, Matsaganis MD (2013). How interpersonal communication mediates the relationship of multichannel communication connections to health-enhancing and health-threatening behaviors. J Health Commun.

[77] Quittschalle J, Stein J, Luppa M, Pabst A, Löbner M, Koenig HH, Riedel-Heller SG (2020). Internet use in old age: results of a German population-representative survey. J Med Internet Res.

[78] Baumann E, Czerwinski F, Reifegerste D (2017). Gender-specific determinants and patterns of online health information seeking: results from a representative German health survey. J Med Internet Res.

[79] Bidmon S, Terlutter R (2015). Gender differences in searching for health information on the internet and the virtual patient-physician relationship in Germany: exploratory results on how men and women differ and why. J Med Internet Res.

[80] Xiong Z, Zhang L, Li Z, Xu W, Zhang Y, Ye T (2021). Frequency of online health information seeking and types of information sought among the general Chinese population: cross-sectional study. J Med Internet Res.

[81] Results of the 2017 employment structure basic survey. Statistics Bureau and Ministry of Internal Affairs and Communications, Japan.

[82] Comprehensive survey of living conditions 2017. Ministry of Health, Labour and Welfare.

[83] Scharkow M (2016). The accuracy of self-reported internet use—a validation study using client log data. Commun Methods Meas.

[84] Boase J, Ling R (2013). Measuring mobile phone use: self-report versus log data. J Comput Mediat Commun.

[85] Prior M (2009). The immensely inflated news audience: assessing bias in self-reported news exposure. Public Opin Q.

[86] Almenara CA, Machackova H, Smahel D (2019). Sociodemographic, attitudinal, and behavioral correlates of using nutrition, weight loss, and fitness websites: an online survey. J Med Internet Res.

[87] Park MB, Wang JM, Bulwer BE (2021). Global dieting trends and seasonality: social big-data analysis may be a useful tool. Nutrients.

[88] Madden KM (2017). The seasonal periodicity of healthy contemplations about exercise and weight loss: ecological correlational study. JMIR Public Health Surveill.

[89] Beck F, Richard J, Nguyen-Thanh V, Montagni I, Parizot I, Renahy E (2014). Use of the internet as a health information resource among French young adults: results from a nationally representative survey. J Med Internet Res.

[90] Young R (1996). The household context for women's health care decisions: impacts of U.K. policy changes. Soc Sci Med.

[91] Yoo E, Robbins LS (2008). Understanding middle‐aged women's health information seeking on the web: a theoretical approach. J Am Soc Inf Sci Technol.

[92] Kininmonth AR, Jamil N, Almatrouk N, Evans CE (2017). Quality assessment of nutrition coverage in the media: a 6-week survey of five popular UK newspapers. BMJ Open.

[93] Kamiński M, Skonieczna-Żydecka K, Nowak JK, Stachowska E (2020). Global and local diet popularity rankings, their secular trends, and seasonal variation in Google Trends data. Nutrition.

[94] Helm J, Jones RM (2016). Practice paper of the academy of nutrition and dietetics: social media and the dietetics practitioner: opportunities, challenges, and best practices. J Acad Nutr Diet.

[95] Eysenbach G, Köhler C (2002). How do consumers search for and appraise health information on the world wide web? Qualitative study using focus groups, usability tests, and in-depth interviews. BMJ.

[96] Halpern DF (1998). Teaching critical thinking for transfer across domains: disposition, skills, structure training, and metacognitive monitoring. Am Psychol.

