# Supplementary material for: Prevalence and Correlates of Dietary and Nutrition Information Seeking Through Various Web-Based and Offline Media Sources Among Japanese Adults: Web-Based Cross-Sectional Study
Source: JMIR Public Health Surveill. 2024 Feb 14;10:e54805. doi: 10.2196/54805 (PMC10902774; doi:10.2196/54805)
Supplement: Multimedia Appendix 1 [file publichealth_v10i1e54805_app1.docx]

Multimedia Appendix 1: Detailed description of the assessment of food literacy.

The self-perceived food literacy (SPFL) was assessed using the Japanese version of the SPFL scale. First, the original SPFL scale, which was developed in Dutch with the English translation available [49], was translated into Japanese by the second author. The Japanese translation was checked, and when necessary modified, by the first author. Then, back translation (to English) was conducted using DeepL Translator, a neural machine translation service. The first and second authors further checked and then approved the Japanese version and its back-translated version. Finally, the backward translation was reviewed by the original researcher [49], based on which relevant modifications were made such that the translated version better reflected the original scale.

The SPFL scale is an expert-based and theory-driven tool for measuring food literacy with respect to healthy eating [49]. The validity of the original Dutch version has been described elsewhere [49]. The SPFL scale, consisting of 29 items, measures 8 domains of food literacy: food preparation skills (6 items), resilience and resistance (6 items), healthy snack styles (4 items), social and conscious eating (3 items), examining food labels (2 items), daily food planning (2 items), healthy budgeting (2 items), and healthy food stockpiling (4 items) [49]. The questionnaire (in English) has been described in full elsewhere [49]. Participants were asked to answer all the questions based on a 5-point Likert scale (1 = *not at all* or *never*; 5 = *yes* or *always*). The total score was calculated as the average of all the items, with negative items reversed, indicating that the higher the score, the higher food literacy is (possible scores ranging from 1 to 5) [49]. The score for each domain was also calculated as the sum of the scores divided by the number of items. In the present study population (N=5998), the Cronbach’s alpha for the assessment of internal consistency was 0.80 for total score, 0.88 for food preparation skills, 0.65 for resilience and resistance, 0.69 for healthy snack styles, 0.47 for social and conscious eating, 0.90 for examining food labels, 0.76 for daily food planning, 0.83 for healthy budgeting, and 0.77 for healthy food stockpiling, which was considered generally good or adequate (except for social and conscious eating) but also comparable to observations in Dutch adults [49].
