# Supplementary material for: Prevalence and Correlates of Dietary and Nutrition Information Seeking Through Various Web-Based and Offline Media Sources Among Japanese Adults: Web-Based Cross-Sectional Study
Source: JMIR Public Health Surveill. 2024 Feb 14;10:e54805. doi: 10.2196/54805 (PMC10902774; doi:10.2196/54805)

Multimedia Appendix 2: Detailed description of the assessment of diet quality.

In the present study, we used the Healthy Eating Index (HEI)-2020 [50] as a measure of diet quality. The HEI-2020 is an established, 100-point scale to assess compliance with the 2020–2025 Dietary Guidelines for Americans [2], with a higher score indicating a better quality of overall diet. The HEI-2020 consists of nine adequacy components (total fruits, whole fruits, total vegetables, greens and beans, whole grains, dairy products, total protein foods, seafood and plant proteins, and fatty acids as the ratio of the sum of polyunsaturated fatty acids and monounsaturated fatty acids to saturated fatty acids) and four moderation components (refined grains, sodium, added sugars, and saturated fats). The efficacy of the HEI-2015, which completely aligns with the HEI-2020 [50], in assessing the overall diet quality of Japanese has been supported by our previous analyses: a higher total score in the HEI-2015 was associated with favorable patterns of the overall diet, including higher intakes of dietary fiber and key vitamins and minerals and lower intakes of saturated fats, added sugars, and sodium [59,60].

Information on dietary habits during the preceding month was collected using the short version of Meal-based Diet History Questionnaire (hereafter referred to as sMDHQ). Briefly, the original Meal-based Diet History Questionnaire (MDHQ) consists of three parts: (i) consumption frequency of major food groups for each of main meals (breakfast, lunch, and dinner) and snacks (morning snack, afternoon snack, and night snack) separately (113 questions); (ii) relative consumption frequency of sub-food groups within the major food groups (72 questions) with questions on consumption frequency and portion size for alcoholic beverages (10 questions); (iii) general eating behaviors (22 questions) [51,52,61]. In contrast, as shown in Figure below, the sMDHQ asks only about consumption frequency of major food groups (except for non-caloric beverages) for the main meals (derived from Part 1 of the MDHQ; 66 questions) and alcoholic beverages (derived from Part 2 of the MDHQ; 10 questions).

Prior to the present study, the validity of sMDHQ was examined using a simulated analysis using a different dataset. The HEI-2020 was calculated following simulation procedures for 111 females and 111 males who completed both the MDHQ and a 4-non-consecutive-day weighed dietary record [52,61]. In the simulation, items deleted from Part 1 were deemed as no-consumption for all individuals, while the intermediate response was used for all individuals for items from Part 2 and Part 3 (see Figure below). We then examined the correlation between the HEI-2020 obtained by this simulation procedure and that derived from the dietary record [52,61]. Spearman correlation coefficients between the HEI-2020 derived from the sMDHQ and that from the dietary record for overall diet was 0.47 for females and 0.62 for males. These results suggest that the sMDHQ is comparable with the MDHQ in terms of the ability to rank individuals according to the quality of the overall diet [52].

In the present study, the HEI-2020 was calculated based on the estimates of dietary intake derived from the sMDHQ. Component scores needed to calculate HEI-2020 were calculated using the Japanese version [59] of the US Food Patterns Equivalents Database [62], except for fatty acids and sodium, for which the 2015 version of the Standard Tables of Food Composition in Japan [63] was used. As described in detail elsewhere [59], we calculated the HEI-2020 component scores based on energy-adjusted values of dietary intake, namely amount per 1000 kcal of energy or percentage of energy, except for fatty acids, and then summed these scores to obtain the HEI-2020 score. These calculations were done for each meal type, and the score for overall diet was calculated using the sum of the intake of each meal type.

Figure. Summary of the short version of the Meal-based Diet History Questionnaire. MDHQ, Meal-based Diet History Questionnaire; sMDHQ, short version of MDHQ


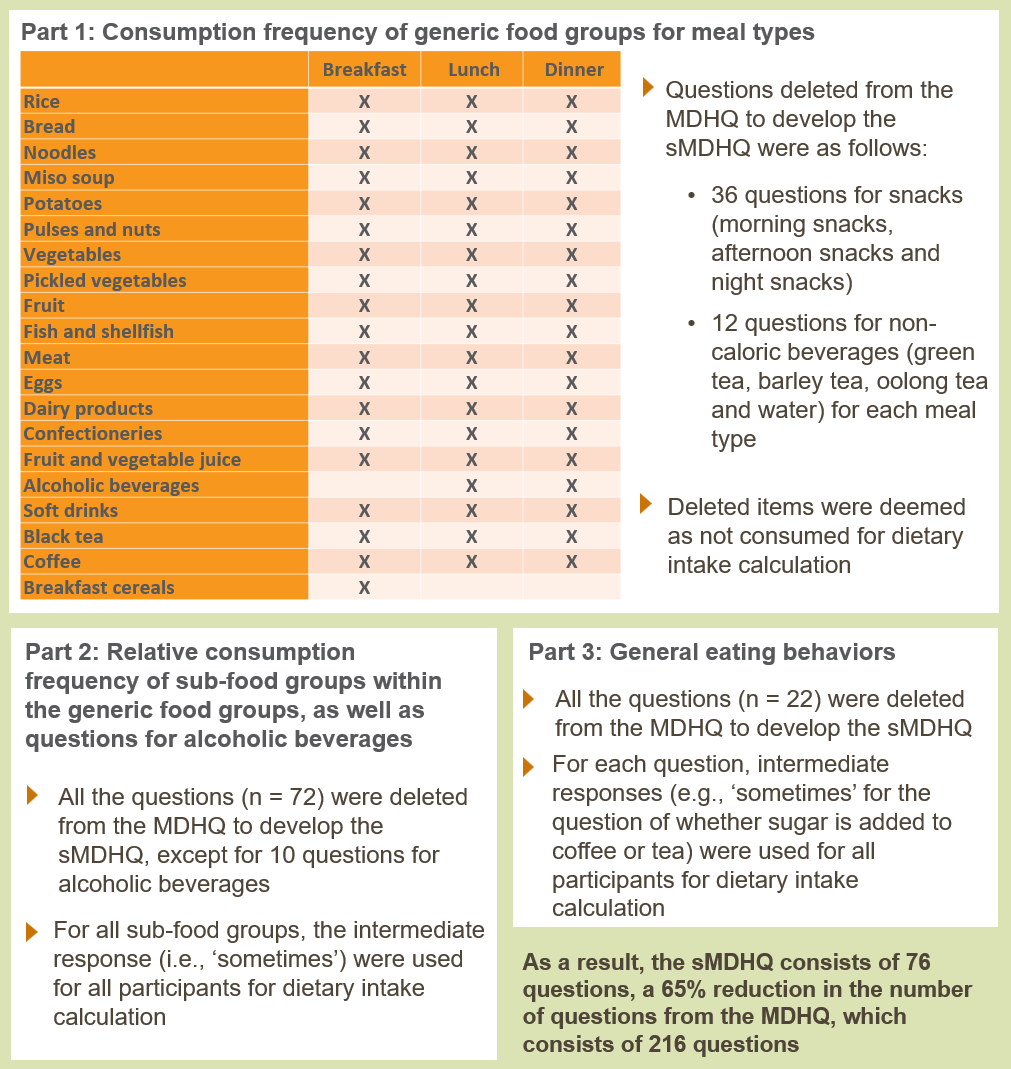

Supplement: Multimedia Appendix 2 [file publichealth_v10i1e54805_app2.docx]
