# Supplementary material for: Prevalence and Correlates of Dietary and Nutrition Information Seeking Through Various Web-Based and Offline Media Sources Among Japanese Adults: Web-Based Cross-Sectional Study
Source: JMIR Public Health Surveill. 2024 Feb 14;10:e54805. doi: 10.2196/54805 (PMC10902774; doi:10.2196/54805)
Supplement: Multimedia Appendix 3 [file publichealth_v10i1e54805_app3.docx]

Multimedia Appendix 3: Associations among the top 6 media sources used by Japanese adults for seeking diet and nutrition information (N=5998).^a^

|  |  | Television | | Web searches | | Websites of government and medical manufacturers | | Newspapers | | Books and magazines | | Video sites  (eg, YouTube) | |
| --- | --- | --- | --- | --- | --- | --- | --- | --- | --- | --- | --- | --- | --- |
|  | N | n | % | n | % | n | % | n | % | n | % | n | % |
| Overall | 5998 | 1973 | 32.9 | 1333 | 22.2 | 997 | 16.6 | 901 | 15.0 | 697 | 11.6 | 634 | 10.6 |
| Television |  |  |  |  |  |  |  |  |  |  |  |  |  |
| No | 4025 | 0 | 0 | 578 | 14.4 | 575 | 14.3 | 303 | 7.5 | 332 | 8.2 | 307 | 7.6 |
| Yes | 1973 | 1973 | 100 | 755 | 38.3 | 422 | 21.4 | 598 | 30.3 | 365 | 18.5 | 327 | 16.6 |
| Web searches |  |  |  |  |  |  |  |  |  |  |  |  |  |
| No | 4665 | 1218 | 26.1 | 0 | 0 | 603 | 12.9 | 632 | 13.5 | 456 | 9.8 | 282 | 6.0 |
| Yes | 1333 | 755 | 56.6 | 1333 | 100 | 394 | 29.6 | 269 | 20.2 | 241 | 18.1 | 352 | 26.4 |
| Websites of government and medical  manufacturers |  |  |  |  |  |  |  |  |  |  |  |  |  |
| No | 5001 | 1551 | 31.0 | 939 | 18.8 | 0 | 0 | 669 | 13.4 | 437 | 8.7 | 459 | 9.2 |
| Yes | 997 | 422 | 42.3 | 394 | 39.5 | 997 | 100 | 232 | 23.3 | 260 | 26.1 | 175 | 17.6 |
| Newspapers |  |  |  |  |  |  |  |  |  |  |  |  |  |
| No | 5097 | 1375 | 27.0 | 1064 | 20.9 | 765 | 15.0 | 0 | 0 | 455 | 8.9 | 531 | 10.4 |
| Yes | 901 | 598 | 66.4 | 269 | 29.9 | 232 | 25.7 | 901 | 100 | 242 | 26.9 | 103 | 11.4 |
| Books and magazines |  |  |  |  |  |  |  |  |  |  |  |  |  |
| No | 5301 | 1608 | 30.3 | 1092 | 20.6 | 737 | 13.9 | 659 | 12.4 | 0 | 0 | 527 | 9.9 |
| Yes | 697 | 365 | 52.4 | 241 | 34.6 | 260 | 37.3 | 242 | 34.7 | 697 | 100 | 107 | 15.4 |
| Video sites (eg, YouTube) |  |  |  |  |  |  |  |  |  |  |  |  |  |
| No | 5364 | 1646 | 30.7 | 988 | 18.4 | 822 | 15.3 | 798 | 14.9 | 590 | 11.0 | 0 | 0 |
| Yes | 634 | 327 | 51.6 | 352 | 55.5 | 175 | 27.6 | 103 | 16.2 | 107 | 16.9 | 634 | 100 |

^a^All associations were statistically significant based on the chi-square test (*P* <.0001), except for the association between newspapers and video sites (*P*=.36).
