# Supplementary material for: Prevalence and Correlates of Dietary and Nutrition Information Seeking Through Various Web-Based and Offline Media Sources Among Japanese Adults: Web-Based Cross-Sectional Study
Source: JMIR Public Health Surveill. 2024 Feb 14;10:e54805. doi: 10.2196/54805 (PMC10902774; doi:10.2196/54805)
Supplement: Multimedia Appendix 4 [file publichealth_v10i1e54805_app4.docx]

Multimedia Appendix 4: Prevalence of diet and nutrition information seeking through the top 6 media sources among Japanese adults, according to participant characteristics (N=5998).^a^

|  | N | Television | Web searches | Websites of government and medical manufacturers | Newspapers | Books and magazines | Video sites (eg, YouTube) |
| --- | --- | --- | --- | --- | --- | --- | --- |
| Sex |  |  |  |  |  |  |  |
| Male | 2687 | 29.4 | 22.2 | 16.0 | 17.4 | 9.3 | 11.8 |
| Female | 3311 | 35.8 | 22.2 | 17.1 | 13.1 | 13.5 | 9.6 |
| *P* value |  | <.0001 | .99 | .28 | <.0001 | <.0001 | .007 |
| Age (years) |  |  |  |  |  |  |  |
| 20 to 39 | 2273 | 30.2 | 20.3 | 18.9 | 6.0 | 12.1 | 14.0 |
| 40 to 59 | 2279 | 32.4 | 24.0 | 16.6 | 14.9 | 11.2 | 9.0 |
| 60 to 79 | 1446 | 37.9 | 22.5 | 13.1 | 29.4 | 11.5 | 7.7 |
| *P* value |  | <.0001 | .01 | <.0001 | <.0001 | .68 | <.0001 |
| Weight status^b^ |  |  |  |  |  |  |  |
| Underweight | 783 | 33.3 | 25.2 | 18.4 | 14.3 | 11.5 | 10.1 |
| Normal weight | 4171 | 33.4 | 22.1 | 16.7 | 15.6 | 11.9 | 10.9 |
| Overweight | 1044 | 30.7 | 20.7 | 15.0 | 13.4 | 10.6 | 9.6 |
| *P* value |  | .24 | .07 | .16 | .18 | .52 | .41 |
| Education level |  |  |  |  |  |  |  |
| Junior high or high school | 951 | 38.6 | 23.9 | 10.5 | 16.5 | 8.6 | 13.4 |
| Junior college or technical  school | 1394 | 34.3 | 23.7 | 14.6 | 11.4 | 11.7 | 11.3 |
| University or higher | 3623 | 31.0 | 21.3 | 19.0 | 16.0 | 12.4 | 9.6 |
| Other | 30 | 16.7 | 10.0 | 13.3 | 16.7 | 10.0 | 10.0 |
| *P* value |  | <.0001 | .06 | <.0001 | .0003 | .01 | .006 |
| Household income^c^ |  |  |  |  |  |  |  |
| <4 million Japanese yen | 1130 | 33.2 | 23.5 | 14.4 | 15.9 | 10.8 | 11.4 |
| 4 to 7 million Japanese yen | 1596 | 34.1 | 24.7 | 16.6 | 15.0 | 12.0 | 11.7 |
| >7 million Japanese yen | 2297 | 33.7 | 22.1 | 19.3 | 15.7 | 13.0 | 10.8 |
| Unknown or do not want to  answer | 975 | 28.6 | 17.0 | 12.9 | 12.4 | 8.7 | 7.3 |
| *P* value |  | .02 | <.0001 | <.0001 | .08 | .004 | .003 |
| Employment status |  |  |  |  |  |  |  |
| None | 1010 | 38.5 | 23.9 | 12.9 | 23.3 | 10.5 | 10.5 |
| Student | 87 | 35.6 | 16.1 | 23.0 | 6.9 | 17.2 | 21.8 |
| Part-time job | 1025 | 37.0 | 23.3 | 13.2 | 16.2 | 12.1 | 10.5 |
| Full-time job | 3876 | 30.3 | 21.6 | 18.4 | 12.7 | 11.7 | 10.3 |
| *P* value |  | <.0001 | .18 | <.0001 | <.0001 | .25 | .008 |
| Marital status |  |  |  |  |  |  |  |
| Unmarried | 2305 | 29.1 | 22.3 | 18.0 | 9.6 | 12.1 | 12.6 |
| Married | 3629 | 35.5 | 22.3 | 16.0 | 18.6 | 11.4 | 9.3 |
| Do not want to answer | 64 | 20.3 | 10.9 | 6.3 | 6.3 | 9.4 | 10.9 |
| *P* value |  | <.0001 | .09 | .01 | <.0001 | .62 | .0003 |
| Living alone |  |  |  |  |  |  |  |
| No | 4682 | 34.6 | 22.0 | 16.5 | 16.8 | 11.5 | 10.0 |
| Yes | 1316 | 26.7 | 23.1 | 17.0 | 8.7 | 12.0 | 12.6 |
| *P* value |  | <.0001 | .39 | .66 | <.0001 | .62 | .006 |
| Presence of chronic disease |  |  |  |  |  |  |  |
| No | 3849 | 32.4 | 21.7 | 16.0 | 12.4 | 11.3 | 10.9 |
| Yes | 2149 | 33.8 | 23.2 | 17.7 | 19.6 | 12.2 | 9.9 |
| *P* value |  | .25 | .19 | .09 | <.0001 | .30 | .22 |
| Smoking status |  |  |  |  |  |  |  |
| Never | 3886 | 33.7 | 21.8 | 17.6 | 14.3 | 12.7 | 9.7 |
| Past | 1213 | 31.7 | 23.3 | 15.8 | 18.5 | 11.0 | 11.7 |
| Current | 899 | 30.9 | 22.6 | 13.5 | 13.5 | 7.8 | 12.7 |
| *P* value |  | .17 | .51 | .008 | .0007 | .0002 | .01 |
| Region |  |  |  |  |  |  |  |
| Hokkaido and Tohoku | 608 | 32.1 | 21.9 | 16.6 | 16.0 | 12.3 | 12.3 |
| Kanto | 2377 | 31.9 | 23.1 | 17.3 | 13.6 | 12.0 | 10.4 |
| Tokai and Hokuriku | 901 | 33.7 | 22.9 | 15.8 | 15.5 | 11.9 | 10.1 |
| Kinki | 1072 | 32.4 | 21.6 | 16.4 | 15.0 | 11.0 | 9.3 |
| Chugoku and Shikoku | 475 | 36.2 | 21.7 | 15.8 | 19.0 | 11.4 | 11.2 |
| Kyushu | 565 | 35.0 | 19.8 | 16.1 | 15.8 | 10.4 | 12.0 |
| *P* value |  | .38 | .63 | .89 | .07 | .88 | .36 |
| Municipality level |  |  |  |  |  |  |  |
| Ward | 2300 | 33.3 | 22.0 | 16.8 | 14.6 | 12.4 | 10.5 |
| City | 3267 | 32.8 | 22.9 | 16.4 | 15.3 | 11.1 | 10.5 |
| Town and village | 321 | 32.7 | 18.7 | 18.4 | 17.1 | 11.5 | 11.5 |
| Missing | 110 | 27.3 | 19.1 | 14.6 | 10.0 | 11.8 | 12.7 |
| *P* value |  | .62 | .28 | .75 | .28 | .47 | .82 |
| Nutrition- and health-related  occupation |  |  |  |  |  |  |  |
| None (ie, general public) | 3021 | 36.7 | 23.7 | 13.3 | 17.3 | 9.6 | 11.9 |
| Nongovernmental qualification  related to food and nutrition | 504 | 26.0 | 20.4 | 15.9 | 12.5 | 14.9 | 16.3 |
| Media | 359 | 39.8 | 20.3 | 13.1 | 23.4 | 14.2 | 6.7 |
| Dietitian and registered dietitian | 631 | 26.0 | 20.1 | 31.5 | 10.0 | 21.7 | 7.4 |
| Physician and dentist | 602 | 23.6 | 22.4 | 19.4 | 15.3 | 10.1 | 9.6 |
| Other health professional | 881 | 32.2 | 20.4 | 17.1 | 8.5 | 9.3 | 7.2 |
| *P* value |  | <.0001 | .13 | <.0001 | <.0001 | <.0001 | <.0001 |
| Health literacy score |  |  |  |  |  |  |  |
| Quartile 1 (1.0 to 3.0) | 1459 | 21.2 | 12.3 | 7.5 | 8.2 | 5.6 | 6.4 |
| Quartile 2 (3.2 to 3.6) | 1746 | 31.9 | 19.1 | 12.4 | 13.1 | 8.4 | 9.9 |
| Quartile 3 (3.8 to 3.8) | 844 | 35.9 | 23.7 | 18.5 | 17.9 | 13.3 | 9.5 |
| Quartile 4 (4.0 to 5.0) | 1949 | 41.3 | 31.8 | 26.4 | 20.6 | 18.4 | 14.7 |
| *P* value |  | <.0001 | <.0001 | <.0001 | <.0001 | <.0001 | <.0001 |
| Food literacy score |  |  |  |  |  |  |  |
| Quartile 1 (1.52 to 2.90) | 1499 | 33.1 | 20.4 | 10.2 | 11.0 | 5.7 | 9.0 |
| Quartile 2 (2.91 to 3.14) | 1514 | 28.5 | 18.4 | 10.4 | 11.9 | 7.5 | 8.7 |
| Quartile 3 (3.15 to 3.45) | 1474 | 34.8 | 25.2 | 19.4 | 16.8 | 13.4 | 11.6 |
| Quartile 4 (3.46 to 4.76) | 1511 | 35.2 | 25.0 | 26.5 | 20.4 | 19.8 | 13.0 |
| *P* value |  | .0003 | <.0001 | <.0001 | <.0001 | <.0001 | <.0001 |
| Healthy Eating Index-2020 |  |  |  |  |  |  |  |
| Quartile 1 (23.1 to 45.7) | 1499 | 30.1 | 20.1 | 10.4 | 10.3 | 5.5 | 9.9 |
| Quartile 2 (45.8 to 50.4) | 1500 | 32.5 | 22.3 | 15.7 | 13.5 | 9.3 | 11.3 |
| Quartile 3 (50.5 to 55.3) | 1500 | 34.5 | 21.3 | 18.1 | 15.3 | 12.5 | 9.9 |
| Quartile 4 (55.4 to 76.5) | 1499 | 34.5 | 25.2 | 22.3 | 20.9 | 19.1 | 11.3 |
| *P* value |  | .03 | .007 | <.0001 | <.0001 | <.0001 | .37 |

^a^*P* values are based on chi-square test.

^b^Underweight, normal weight, and overweight were defined having BMIs of <18.5, ≥18.5 to <25, and ≥25 kg/m^2^, respectively.

^c^US $1=JPY 148.22.
