# Supplementary material for: Prevalence and Correlates of Dietary and Nutrition Information Seeking Through Various Web-Based and Offline Media Sources Among Japanese Adults: Web-Based Cross-Sectional Study
Source: JMIR Public Health Surveill. 2024 Feb 14;10:e54805. doi: 10.2196/54805 (PMC10902774; doi:10.2196/54805)
Supplement: Multimedia Appendix 6 [file publichealth_v10i1e54805_app6.docx]

Multimedia Appendix 6: Associations of participant characteristics with diet and nutrition information seeking through the top 6 media sources among Japanese adults with nutrition and health-related occupations (2977/5998, 49.63%).^a^

|  | Television | | Web searches | | Websites of government and medical manufacturers | | Newspapers | | Books and magazines | | Video sites (eg, YouTube) | |
| --- | --- | --- | --- | --- | --- | --- | --- | --- | --- | --- | --- | --- |
|  | OR^b^ | 95% CI | OR^c^ | 95% CI | OR^d^ | 95% CI | OR^e^ | 95% CI | OR^f^ | 95% CI | OR^g^ | 95% CI |
| Female sex (ref: male) | **1.65** | **1.28, 2.14** | 0.79 | 0.60, 1.05 | 1.01 | 0.76, 1.34 | **0.56** | **0.39, 0.81** | 1.01 | 0.72, 1.42 | **0.65** | **0.45, 0.94** |
| Age (per 1-year increment) | 1.01 | 0.999, 1.02 | **1.01** | **1.003, 1.02** | **0.98** | **0.97, 0.99** | **1.06** | **1.05, 1.07** | 0.99 | 0.98, 1.01 | **0.96** | **0.95, 0.98** |
| Weight status (ref: normal weight)^h^ |  |  |  |  |  |  |  |  |  |  |  |  |
| Underweight | 1.12 | 0.86, 1.47 | 1.32 | 0.99, 1.76 | 1.03 | 0.77, 1.38 | 1.25 | 0.85, 1.84 | **0.65** | **0.45, 0.94** | 0.91 | 0.60, 1.38 |
| Overweight | 0.95 | 0.73, 1.24 | 0.82 | 0.61, 1.09 | 1.07 | 0.80, 1.44 | 0.82 | 0.58, 1.17 | 1.13 | 0.80, 1.60 | 0.88 | 0.58, 1.32 |
| Education level (ref: junior high or high school) |  |  |  |  |  |  |  |  |  |  |  |  |
| Junior college or technical school | 1.07 | 0.64, 1.79 | 1.25 | 0.71, 2.21 | 1.31 | 0.63, 2.71 | 1.12 | 0.57, 2.19 | 1.10 | 0.55, 2.21 | **0.50** | **0.26, 0.94** |
| University or higher | 0.88 | 0.54, 1.45 | 0.97 | 0.56, 1.66 | 1.89 | 0.94, 3.82 | 1.51 | 0.81, 2.82 | 1.14 | 0.58, 2.21 | **0.42** | **0.23, 0.75** |
| Other | <0.001 | <0.001, >999 | 1.50 | 0.26, 8.78 | 2.56 | 0.44, 15.02 | **6.59** | **1.12, 38.75** | 3.56 | 0.72, 17.54 | 0.95 | 0.10, 8.67 |
| Household income (ref: <4 million Japanese yen)^i^ |  |  |  |  |  |  |  |  |  |  |  |  |
| 4 to 7 million Japanese yen | 0.79 | 0.57, 1.10 | 1.23 | 0.86, 1.76 | 0.93 | 0.65, 1.34 | 0.93 | 0.58, 1.51 | 1.21 | 0.80, 1.84 | 0.74 | 0.46, 1.19 |
| >7 million Japanese yen | 1.02 | 0.73, 1.44 | 0.90 | 0.62, 1.31 | 0.87 | 0.60, 1.27 | 0.80 | 0.49, 1.29 | 1.18 | 0.77, 1.82 | 0.93 | 0.57, 1.51 |
| Unknown or do not want to answer | 0.82 | 0.56, 1.20 | 0.76 | 0.49, 1.16 | 0.80 | 0.52, 1.21 | 0.88 | 0.52, 1.50 | 0.98 | 0.60, 1.60 | 0.88 | 0.51, 1.55 |
| Employment status (ref: none) |  |  |  |  |  |  |  |  |  |  |  |  |
| Student | 1.83 | 0.46, 7.30 | 0.38 | 0.06, 2.45 | 2.14 | 0.46, 9.90 | 1.90 | 0.17, 21.42 | 1.01 | 0.16, 6.46 | 2.13 | 0.45, 9.98 |
| Part-time job | 0.74 | 0.38, 1.43 | 1.13 | 0.54, 2.40 | 0.76 | 0.33, 1.75 | **3.35** | **1.22, 9.22** | 1.11 | 0.47, 2.65 | 1.54 | 0.56, 4.26 |
| Full-time job | 0.66 | 0.34, 1.26 | 1.07 | 0.51, 2.25 | 1.09 | 0.48, 2.49 | **3.15** | **1.16, 8.57** | 1.38 | 0.59, 3.25 | 1.45 | 0.54, 3.92 |
| Marital status (ref: unmarried) |  |  |  |  |  |  |  |  |  |  |  |  |
| Married | 1.15 | 0.89, 1.47 | 0.98 | 0.74, 1.28 | 1.02 | 0.78, 1.34 | 0.92 | 0.64, 1.32 | 0.99 | 0.72, 1.36 | 0.86 | 0.59, 1.25 |
| Do not want to answer | 0.66 | 0.24, 1.82 | 0.84 | 0.28, 2.48 | 0.55 | 0.16, 1.87 | 0.77 | 0.17, 3.53 | 1.30 | 0.43, 3.92 | 1.64 | 0.49, 5.44 |
| Living alone (ref: no) | **0.60** | **0.45, 0.81** | 1.02 | 0.74, 1.38 | 0.99 | 0.72, 1.35 | **0.59** | **0.38, 0.91** | 1.10 | 0.76, 1.58 | 1.19 | 0.78, 1.82 |
| Presence of chronic disease (ref: no) | 0.84 | 0.68, 1.04 | 0.95 | 0.76, 1.19 | **1.51** | **1.20, 1.88** | 0.86 | 0.65, 1.13 | 0.96 | 0.74, 1.26 | 1.20 | 0.88, 1.64 |
| Smoking status (ref: never) |  |  |  |  |  |  |  |  |  |  |  |  |
| Past | 0.91 | 0.70, 1.18 | 0.85 | 0.64, 1.12 | 0.99 | 0.74, 1.32 | 0.91 | 0.64, 1.28 | 0.87 | 0.61, 1.22 | **1.71** | **1.17, 2.49** |
| Current | 0.98 | 0.74, 1.31 | 0.78 | 0.57, 1.06 | 1.02 | 0.74, 1.41 | 0.92 | 0.64, 1.33 | 0.76 | 0.51, 1.12 | **1.74** | **1.17, 2.57** |
| Region (ref: Kanto) |  |  |  |  |  |  |  |  |  |  |  |  |
| Hokkaido and Tohoku | 1.00 | 0.72, 1.38 | 0.88 | 0.62, 1.25 | 0.98 | 0.69, 1.38 | 1.53 | 0.99, 2.38 | 1.22 | 0.82, 1.82 | 1.10 | 0.68, 1.76 |
| Tokai and Hokuriku | 1.06 | 0.81, 1.39 | 0.91 | 0.68, 1.23 | 0.89 | 0.66, 1.21 | 1.44 | 0.98, 2.11 | 1.08 | 0.76, 1.54 | 1.11 | 0.74, 1.67 |
| Kinki | 0.90 | 0.69, 1.17 | 0.84 | 0.63, 1.12 | 1.02 | 0.77, 1.35 | **1.48** | **1.02, 2.15** | 1.06 | 0.75, 1.49 | 0.89 | 0.58, 1.36 |
| Chugoku and Shikoku | 0.90 | 0.62, 1.28 | 0.95 | 0.65, 1.40 | 0.81 | 0.55, 1.21 | **2.31** | **1.45, 3.68** | 1.09 | 0.69, 1.73 | 1.20 | 0.71, 2.01 |
| Kyushu | 1.29 | 0.95, 1.75 | 0.78 | 0.56, 1.10 | 0.83 | 0.59, 1.17 | **1.86** | **1.24, 2.81** | 1.09 | 0.74, 1.61 | 1.44 | 0.93, 2.24 |
| Municipality level (ref: ward) |  |  |  |  |  |  |  |  |  |  |  |  |
| City | **0.79** | **0.65, 0.96** | 1.23 | 0.996, 1.52 | 1.03 | 0.83, 1.27 | 1.24 | 0.95, 1.62 | 0.83 | 0.65, 1.07 | 1.09 | 0.81, 1.46 |
| Town and village | 0.69 | 0.45, 1.06 | 0.99 | 0.62, 1.60 | 1.43 | 0.92, 2.23 | 1.33 | 0.77, 2.31 | 1.21 | 0.74, 1.98 | 1.32 | 0.72, 2.42 |
| Missing | 0.89 | 0.44, 1.80 | 0.82 | 0.36, 1.90 | 1.03 | 0.48, 2.22 | 0.54 | 0.16, 1.90 | 0.87 | 0.36, 2.07 | 0.90 | 0.34, 2.37 |
| Nutrition- and health-related occupation  (ref: other health professional) |  |  |  |  |  |  |  |  |  |  |  |  |
| Nongovernmental qualification related to food and nutrition | **0.59** | **0.43, 0.81** | 0.99 | 0.70, 1.40 | **0.67** | **0.46, 0.96** | **2.06** | **1.32, 3.21** | **1.69** | **1.12, 2.53** | **2.08** | **1.35, 3.20** |
| Media | 1.28 | 0.93, 1.76 | 0.86 | 0.59, 1.25 | **0.55** | **0.37, 0.83** | **2.34** | **1.51, 3.62** | 1.26 | 0.81, 1.96 | 0.73 | 0.41, 1.30 |
| Dietitian and registered dietitian | **0.61** | **0.46, 0.80** | 1.00 | 0.74, 1.35 | **1.56** | **1.17, 2.08** | 1.20 | 0.79, 1.83 | **1.89** | **1.34, 2.67** | 1.05 | 0.67, 1.65 |
| Physician and dentist | **0.55** | **0.40, 0.76** | 1.22 | 0.87, 1.70 | 0.99 | 0.70, 1.39 | 0.96 | 0.62, 1.47 | 0.91 | 0.59, 1.40 | **1.76** | **1.10, 2.82** |
| Health literacy score (per 1-point increment) | **1.22** | **1.03, 1.44** | **1.48** | **1.23, 1.77** | **1.72** | **1.42, 2.08** | 1.16 | 0.92, 1.46 | **1.51** | **1.22, 1.88** | 1.09 | 0.86, 1.38 |
| Food literacy score (per 1-point increment) | **0.66** | **0.52, 0.85** | 0.92 | 0.70, 1.20 | **2.16** | **1.63, 2.85** | 1.27 | 0.90, 1.79 | **2.12** | **1.52, 2.94** | 1.24 | 0.85, 1.83 |
| Healthy Eating Index-2020 (per 1-point increment) | 1.01 | 0.99, 1.02 | 1.00 | 0.98, 1.01 | 1.02 | 0.999, 1.03 | 1.02 | 0.996, 1.04 | **1.04** | **1.02, 1.05** | 1.00 | 0.98, 1.02 |

CI, confidence interval; OR, odds ratio; ref, reference.

^a^ OR for diet and nutrition information seeking through each media source, in comparison with the reference category of each variable. Statistically significant values shown in bold (*P* <.05).

^b^ Model with diet and nutrition information seeking through television as the dependent variable, and variables listed in the first column and diet and nutrition information seeking through five other media sources (no or yes for each) as the independent variables.

^c^ Model with diet and nutrition information seeking through web searches as the dependent variable, and variables listed in the first column and diet and nutrition information seeking through five other media sources (no or yes for each) as the independent variables.

^d^ Model with diet and nutrition information seeking through websites of government and medical manufacturers as the dependent variable, and variables listed in the first column and diet and nutrition information seeking through five other media sources (no or yes for each) as the independent variables.

^e^ Model with diet and nutrition information seeking through newspapers as the dependent variable, and variables listed in the first column and diet and nutrition information seeking through five other media sources (no or yes for each) as the independent variables.

^f^ Model with diet and nutrition information seeking through books and magazines as the dependent variable, and variables listed in the first column and diet and nutrition information seeking through five other media sources (no or yes for each) as the independent variables.

^g^ Model with diet and nutrition information seeking through video sites (eg, YouTube) as the dependent variable, and variables listed in the first column and diet and nutrition information seeking through five other media sources (no or yes for each) as the independent variables.

^h^ Underweight, normal weight, and overweight were defined having BMIs of <18.5, ≥18.5 to <25, and ≥25 kg/m^2^, respectively.

^i^ US $1=JPY 148.22.
